# Integrating deep learning 3D tracking and biophysical EOD modeling for precise, noninvasive computational neuroethology in freely swimming weakly electric fish

**DOI:** 10.3389/fncom.2026.1810975

**Published:** 2026-06-24

**Authors:** Reynaldo D. Pinto, Caroline G. Forlim, Felipe O. L. da Mota, Helena Pessoni-Figueiredo, Rafael Seixas, Carlos H. M. de Aquino, Lírio O. B. de Almeida, Pablo Varona, Francisco B. Rodriguez

**Affiliations:** 1 Laboratory of Neurobiophysics, São Carlos Institute of Physics (IFSC), University of São Paulo (USP), São Carlos, SP, Brazil; 2 Center for Environmental Neuroscience, Max Planck Institute for Human Development, Berlin, Germany; 3 GNB, Departamento de Ingeniería Informática, Escuela Politécnica Superior, Universidad Autónoma de Madrid, Madrid, Spain

**Keywords:** 3D pose estimation, charge-distribution modeling, electrolocation tracking, EOD spatial simulation, image-charge simulation, kinematic reconstruction, multielectrode recordings, noninvasive electrophysiology

## Abstract

Recording the movement of freely swimming, weakly electric fish during naturalistic behavior and social interactions poses persistent challenges to neuroethologists. Most species are nocturnal; consequently, illumination for video recording often disrupts their natural behavior. Substantial work has addressed this issue using shallow-water tanks, infrared illumination, or indirect movement derivation. While inferring position from a fish’s own electrical signals has also been explored, it remains a promising yet largely unresolved problem in 3D. We introduce an open-source, multimodal, and data-driven method as a first step toward 3D tracking based on electrical signals. Applied to the pulse-type fish *Gymnotus carapo*, this approach links 3D posture to self-generated electric field dynamics. This proof-of-concept study utilized a deep tank, a peripheral electrode array, and a multi-camera system to record a freely swimming animal. Using deep-learning algorithms, we reconstructed 3D skeletal trajectories from video recordings, achieving a spatial accuracy of approximately 0.8 mm per coordinate. Subsequently, we simulated the electric potentials generated by three charge distribution models mapped onto the skeletal structure throughout the tracking period. By comparing these simulations with experimental recordings, we evaluated the consistency of the forward modeling framework and the relative performance of each model. The results suggest that a biologically inspired asymmetric charge distribution better reproduces experimental observations than simpler traditional dipole-like models. Furthermore, we developed a tool dedicated to the precise, non-invasive study of charge distribution along the electric organ. It supports the evaluation of biophysical models linking body posture to electric signal generation. These findings suggest that paired electrical and spatial datasets may support the development of video-free 3D tracking methods based on machine learning for dark, deep-water environments. These results could also assist in the design of novel biomimetic electrolocation systems. Looking ahead, this methodology could be extended to multiple animals and adapted to some other pulse- or wave-type electric fish, thereby offering a framework for integrating anatomy, locomotion, electrosense, and electrogenesis in neuroethological studies.

## 1 Introduction

Weakly electric fish serve as exemplary models for neuroethological research, as their electrical activity can be recorded non-invasively while they navigate freely and engage in complex social behaviors. These animals possess a specialized Electric Organ (EO) that generates Electric Organ Discharges (EODs), establishing an oscillating or pulsed electric field that propagates through the aquatic environment. These fish rely on these self-generated signals for electrolocation, long-range communication, species identification, and foraging—functions fundamental to their survival and reproductive success (Bullock et al., 2005; Caputi, 2025).

The characteristic EOD of a pulse-type weakly electric fish has a complex and characteristic waveform—typically of short duration and consisting of both negative and positive components—when measured by electrodes positioned near the head and tail (Bennett and Grundfest, 1959; Bullock et al., 2005). In *Gymnotus carapo*, this waveform is partitioned into four distinct segments, labeled V1​ through V4​ (Trujillo-Cenóz et al., 1984). Among these, the V3​ and V4​ components are the most prominent and serve as the primary features for our spatial analysis. As these pulses propagate through the water, they can be captured by remote electrode pairs; however, in such cases, the recorded pulse waveform becomes highly dependent on the fish’s proximity and orientation relative to the recording sensor (Forlim and Pinto, 2014). This fundamental relationship between the spatial dynamics of the electric fields and the animal’s position and posture forms the core physical basis of our approach.

Pulse-type weakly electric fish modulate their EOD intervals across various behavioral contexts. Because sensory and motor behaviors are tightly coupled (Hofmann et al., 2014), variations in EOD timing have been correlated with locomotor activity (Forlim and Pinto, 2014), swimming patterns, overall motor behavior (Caputi and Budelli, 1995; von der Emde, 1999; Caputi et al., 2002), and complex social interactions (Matias et al., 2015; Guariento et al., 2016). In neuroethology, integrating the electrical activity of the nervous system with motor behavior is essential for understanding how EOD modulations and motor patterns collectively enable an animal to sense its environment (Hofmann et al., 2014; Skeels et al., 2023). Despite this important factor, accurately tracking the 3D movement of freely swimming fish during exploratory and social behaviors remains a significant challenge. Many behavioral contexts occur exclusively at night or in turbid waters; consequently, even the minimal illumination required for traditional video recording can disrupt natural behaviors. Therefore, the ability to record the position and orientation of these animals in the absence of light offers novel opportunities for neuroethology research.

Previous studies have addressed these tracking challenges in both pulse- and wave-type species using various 2D techniques (Jun et al., 2013, 2014; Forlim and Pinto, 2014; Henninger et al., 2020; Raab et al., 2022; Zlenko et al., 2024; Migliaro et al., 2025). A notable exception is the work of Madhav and colleagues, who successfully tracked the 3D position of the wave-type species *Eigenmannia virescens* (Madhav et al., 2018). Regarding pulse-type Gymnotiformes, Jun et al. (2013, 2014) conducted both video- and EOD-based tracking in shallow-water tanks. Forlim and Pinto (2014) indirectly inferred fish movement dynamics without spatial mapping, and Migliaro et al. (2025) performed horizontal mapping of multiple fish in field experiments using an eight-electrode grid and clustering algorithms. While these methods represent significant advancements, high-fidelity 3D tracking for pulse-type fish remains a largely unresolved problem.

In this study, we describe a methodology to track the 3D position of weakly electric fish in deep water making use of simultaneous recordings of video and EODs. This paired spatial and electrical dataset is designed to facilitate the future development of a tracking framework that eliminates the necessity for video recording, which marks a significant advantage for studying nocturnal behaviors in total darkness. Our approach integrates several complementary experimental and computational techniques. The experimental setup allowed for the synchronized recording of electric signals and multi-angle video of freely swimming fish in a deep-water tank. We employed a deep-learning-based algorithm utilizing the DeepLabCut (Mathis et al., 2018) and Anipose (Karashchuk et al., 2021) toolkits to reconstruct the fish’s 3D skeletal trajectory from video frames. Next, the spatially distributed amplitude of the electric signal was simulated using a simplified biophysical model of the electric organ implemented in Python, utilizing the reconstructed 3D skeletal trajectory as the basis for source charge placement. For that, we modeled the EO by varying the spatial distribution of charges across three distinct theoretical configurations, including the traditional dipole approximation. To account for boundary conditions, we employed the method of images, calculating the electric potential at the specific coordinates of each electrode within a simulated tank of identical dimensions to the experimental setup. Finally, we validated these simulations by comparing the modeled amplitudes against experimental measurements from freely behaving, pulse-type *Gymnotus carapo*. We conclude by discussing the performance, limitations, and future potential of achieving 3D tracking based solely on electric signal recordings.

We consider this approach as a first step toward addressing the inherently ill-posed inverse problem of finding the position and pose of the fish from the electrical recordings alone. The described methodology produces integrated datasets that will allow future machine learning models to extract reliable pose estimations from the electrical field data.

The ability to track a fish’s position based solely on its EODs will provide significant opportunities for both scientific investigation and technological application. This approach will enable neuroethologists to study how fish explore their environment and interact with conspecifics, or with computer-controlled agents that mimic electrical behavior, without disrupting naturalistic patterns, effectively bridging the gap between field-like behavior and controlled laboratory experimentation. Furthermore, precisely determining the timing and duration of movement will help disentangle EOD rate modulations intended for electrolocation (the sensing of physical surroundings) from those intended for electro-communication (the exchange of social behavioral states). Such data will allow for the fine-tuning of computational models simulating signal propagation. These principles can inspire bio-mimetic tracking systems for autonomous underwater vehicles operating in environments where traditional vision is not an option, such as highly turbid waters.

The remainder of the article is structured as follows. The Methods section describes the experimental setup, including the experimental animals, ethical procedures, data acquisition, signal processing, 3D tracking, and biophysical model development and simulation. The Results section presents the analysis of the electrical recordings, the validation of the 3D tracking, and the comparison between simulated and experimental data. Finally, the article concludes with a discussion of the main findings, their implications, and potential future lines of research.

## 2 Materials and methods

### 2.1 Ethics statement

All experimental protocols and procedures were in accordance with the ethical principles of the Society for Neuroscience^1^ and were approved by the Biodiversity Authorization and Information System, Brazilian Institute of Environment and Renewable Natural Resources, Ministry of the Environment and Climate Change, SISBIO/IBAMA/MMA (Authorization 19,446-5) and by the Committee on Ethics in Animal Experimentation of the Physics Institute of São Carlos, University of São Paulo, CEUA/IFSC/USP (Protocol 9,631,130,225).

### 2.2 Animals

Adult specimens, 15–25 cm long, of unsexed *Gymnotus carapo* were acquired from local fishermen in the state of São Paulo, Brazil. The fish were maintained in individual 31.5 L (30 × 35 × 30 cm) unplanted water tanks under continuous filtration, exposed to natural illumination at a controlled temperature (23 ± 1 ⁰C). Each tank was provided with lengths of polyvinyl chloride (PVC) pipe to serve as hiding places. Water conductivity was maintained at 100 ± 5 μS/cm, a value similar to those found in the streams or lagoons where these fish are typically captured. The fish were fed twice a week, primarily with shredded bovine heart and a variety of live food, including earthworms (*Amynthos gracilis* and *Eisenia fetida*) and mealworm larvae (*Tenebrio molitor*). A total of eight fish were used for software adjustments, training and experiments: five fish were video recorded several times while swimming alone freely in several different aquariums. This provided training data for the neural networks into the fish tracking software. Two fish were used to calibrate the apparatus during development; subsequently, data from a single individual (16 cm in length) was acquired and analyzed to demonstrate the developed framework. This final dataset serves as a representative example in our proof-of-concept study and is not intended to provide statistical generalization.

### 2.3 Experimental setup

**Figure 1:**
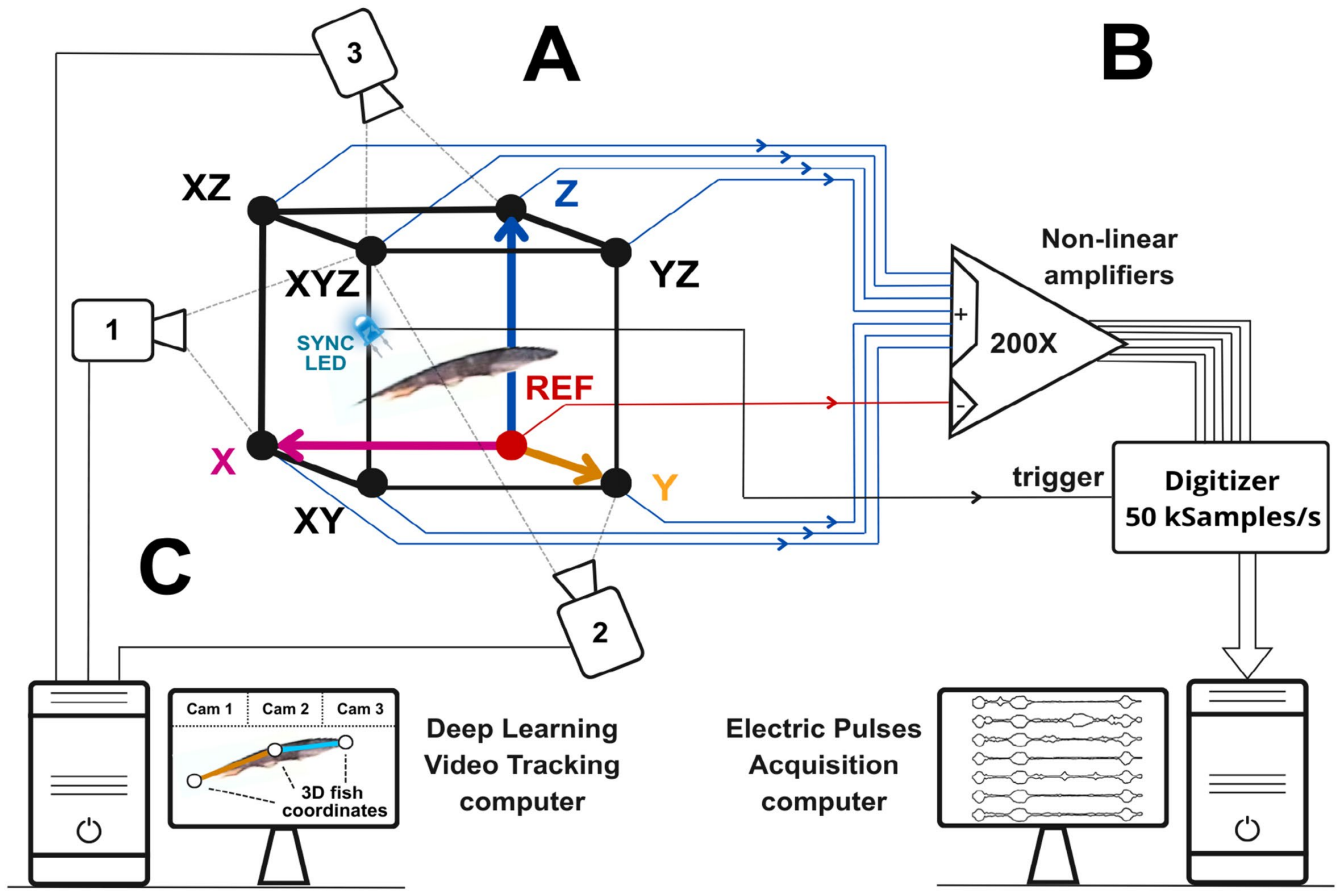
Experimental setup. **(A)** Fish tank: Electric pulses emitted by a freely swimming fish were detected by eight electrodes (“REF,” “X,” “Y,” “Z,” “XY,” “XZ,” “YZ,” and “XYZ”). Electrodes “REF,” “X,” “Y,” and “Z” defined the coordinate system. A synchronization blue LED was activated with a recorded signal at the start of an experiment to synchronize the video and the fish electrical recordings. **(B)** The electrical signals were differentially amplified, digitized, and stored on a computer for subsequent analysis. **(C)** A second computer simultaneously recorded videos from three cameras (1, 2, and 3), arranged at different angles to enable 3D tracking of the fish.

The set-up comprised a deep-water tank, based on previous designs (Forlim and Pinto, 2014; Matias et al., 2015; Guariento et al., 2016), and dual acquisition systems for simultaneous electrical and image recording (Figure 1). The experimental tank (Figure 1A) consisted of a glass rectangular aquarium with a 42 L capacity (inner dimensions: 37 × 37 × 31 cm) filled with water, allowing the fish to swim freely. Three walls of the aquarium were internally lined with matte white plastic to prevent reflections of the fish in the image acquisition system. To minimize noise pickup on the electrical recordings, grounded metallic shielding was positioned around the setup, although the camera system limited full enclosure.

Spatio-temporal electrical signals were measured using a three-dimensional (3D) array of eight electrodes placed at the vertices of the tank (Figure 1A; Forlim and Pinto, 2014; Guariento et al., 2016). Each active electrode consisted of a 0.2 mm diameter stainless steel wire, 2 cm in length, with the wire protruding 1–2 mm into the water through the silicone sealant. These wires were connected to a high-pass filter and a unity-gain buffer (based on a TL071 JFET op-amp), both integrated outside the tank at the point where the steel wire emerges from the sealant. From this stage, the signals were transmitted via shielded cables to custom differential amplifiers for further processing.

Signals from 7 electrodes labeled “X,” “Y,” “Z,” “XY,” “XZ,” “YZ,” and “XYZ”; (Figure 1A) were differentially amplified using the electrode “REF” at the base of the tank, as a common reference. This reference electrode also served as the origin of the orthogonal spatial coordinate system used in the tank. Although this geometry is sub-optimal for common-mode rejection, the use of active electrodes minimized most issues. For this preliminary work, having coincident electrical and spatial references was advantageous for signal interpretation.

To avoid signal saturation as the fish approaches an electrode, custom differential amplifiers (Figure 1B) were developed, featuring nonlinear gain curves: 200x for small-signal inputs and 1.5x for high-amplitude. The time-series data from all differential amplifiers were digitized at a sampling rate of 50 kHz using an analog-to-digital converter (ADC) (Digidata 1322A, Molecular Devices) and stored on a PC-compatible acquisition computer. Data acquisition was triggered by the same signal that activated a blue LED (labeled SYNC LED in Figure 1A), at the beginning of an experiment, to ensure time synchronization between the electrical and video recordings. Following data acquisition, the inverse of the non-linear amplifier gain curves were applied to the recorded time series to reconstruct the original voltage induced by the fish’s electric organ at each electrode.

Three USB cameras (C270 HD, Logitech) were positioned to provide near-orthogonal views of the aquarium (Figure 1C; cameras 1, 2, and 3). Although two cameras are the minimum required for 3D reconstruction via triangulation, three cameras were employed primarily to resolve occlusions due to fish movements. Additionally, this setup increases positional accuracy and provides greater redundancy in data collection. The setup was designed to ensure the SYNC LED (positioned outside the fish’s field of view) was visible to all cameras, facilitating the synchronization of all video and electrical recordings. Furthermore, the configuration prevented fish reflections on non-matte glass surfaces from appearing in the captured frames. Video was recorded simultaneously across all three cameras at a resolution of 1,280 × 960 pixels (4:3 format; 960p) and a frame rate of 30 fps in MP4 format. Video recordings and subsequent computational processing —including deep learning training, three-dimensional reconstruction, and tracking— were conducted on an AMD Ryzen 5 workstation (Figure 1C). The system was equipped with 24 GB of DDR4 RAM, and a NVIDIA RTX 3060 GPU with 12 GB VRAM.

### 2.4 Electrode array experimental data analysis

**Figure 2:**
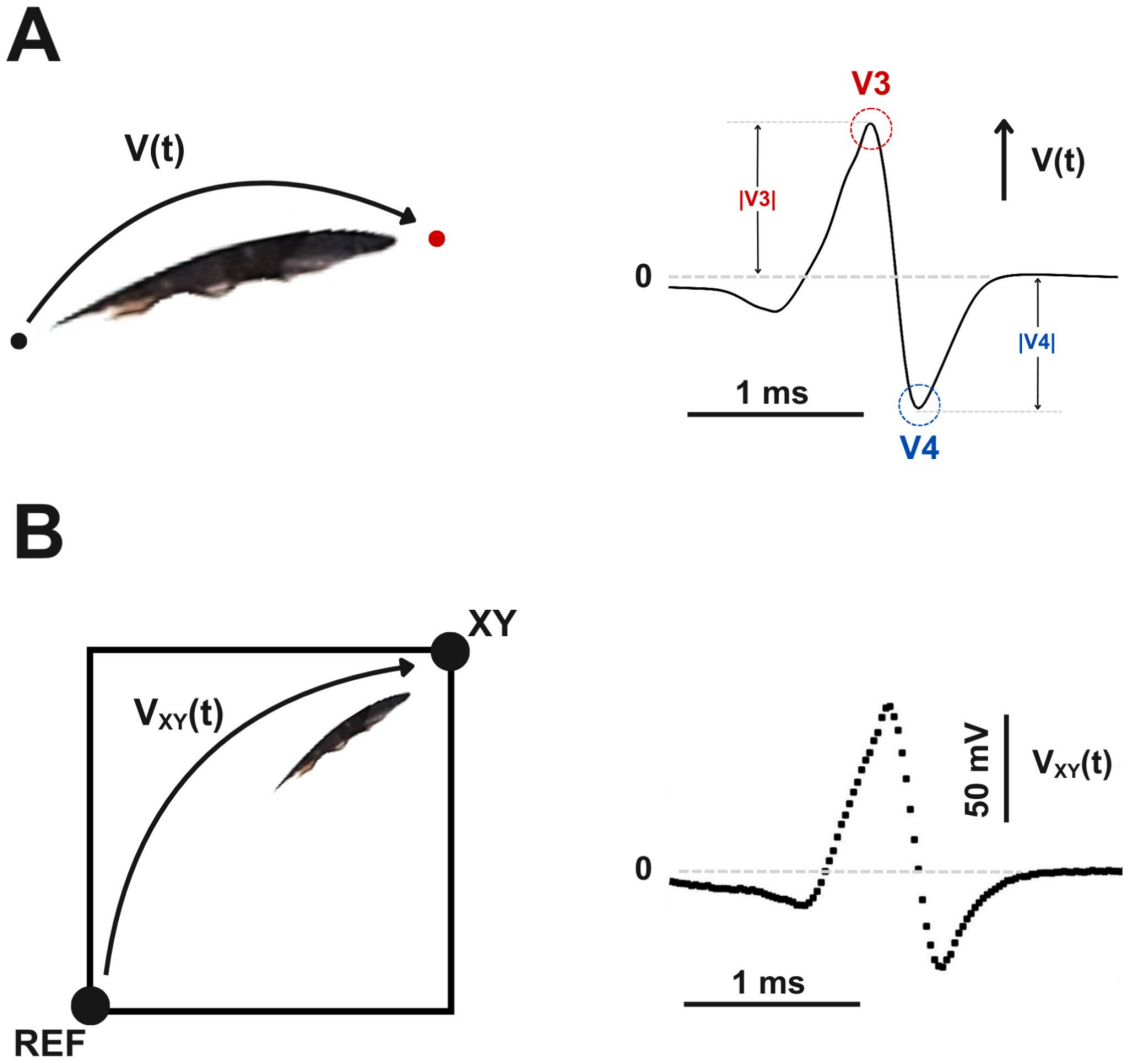
Stereotyped electric organ discharge in *Gymnotus carapo* and the effect of the relative position between fish and electrodes on pulse shape. **(A)** Classic pattern with electrodes positioned near the fish’s body; the corresponding V(*t*) is differentially recorded from the tail (negative) to the head (positive). An asymmetric pulse pattern with several wave components is observed in the right panel. In this configuration, the largest wave components are V3 and V4, with the V4 amplitude being approximately 21% smaller than that of V3. **(B)** Similar recording of VXY(*t*) digitized at 50 kS/s, obtained from two diagonally opposed electrodes at the bottom of the experiment tank. In this case, the fish’s head was oriented toward and significantly closer to electrode “XY” compared to the distance of the tail to the reference electrode “REF.” Both V3 and V4 peaks were well-resolved at this sampling rate, though the pulse asymmetry deviated from the classic pattern shown in **(A)**.

A typical electrical pulse from *Gymnotus carapo* can be observed when a pair of electrodes is placed near the fish (Figure 2A), recording the differential electrical potential V(t) from the tail (negative) to the head (positive). This asymmetric, stereotyped triphasic pulse was generated by the electric organ —distributed along the body length— via the coordinated activation of linearly arrayed electroplaques derived from skeletal muscle (Bennett and Grundfest, 1959). The pulse pattern comprised several wave components well-documented in the literature (Trujillo-Cenóz et al., 1984; Caputi and Budelli, 1995) which occur in a determined sequence: V1, V2, V3, and V4. In this experimental setup, the V3 (positive) and V4 (negative) components predominated. It is important to note that the voltage peaks reflected specific spatial distributions of electric charges within the electric organ that repeated consistently across pulses. In this species, the V4 peak amplitude is approximately 21% smaller than that of V3, resulting in a pulse asymmetry defined as:


Δ%=(∣V4∣−∣V3∣)/∣V3∣=−0.21. (1)

However, when a fish was allowed to swim freely inside a tank with an electrode array as described in Figure 1, the signal captured by a pair of electrodes was no longer stereotyped, but became modulated by the distance and orientation relative to each measurement electrode. Figure 2B displays a linearized signal, V_XY_(t), measured with the fish aligned and its head positioned near electrode XY. An individual pulse is plotted using dots to demonstrate that the acquisition rate of 50 kS/s was sufficient to resolve both the V3 and V4 peaks. Notably, the observed asymmetry exceeded the −21% reported for the classic pattern (Figure 2A), underscoring that the recorded electrical activity was dynamically dependent. It depends on both the fish’s proximity and its orientation relative to each electrode pair.

**Figure 3:**
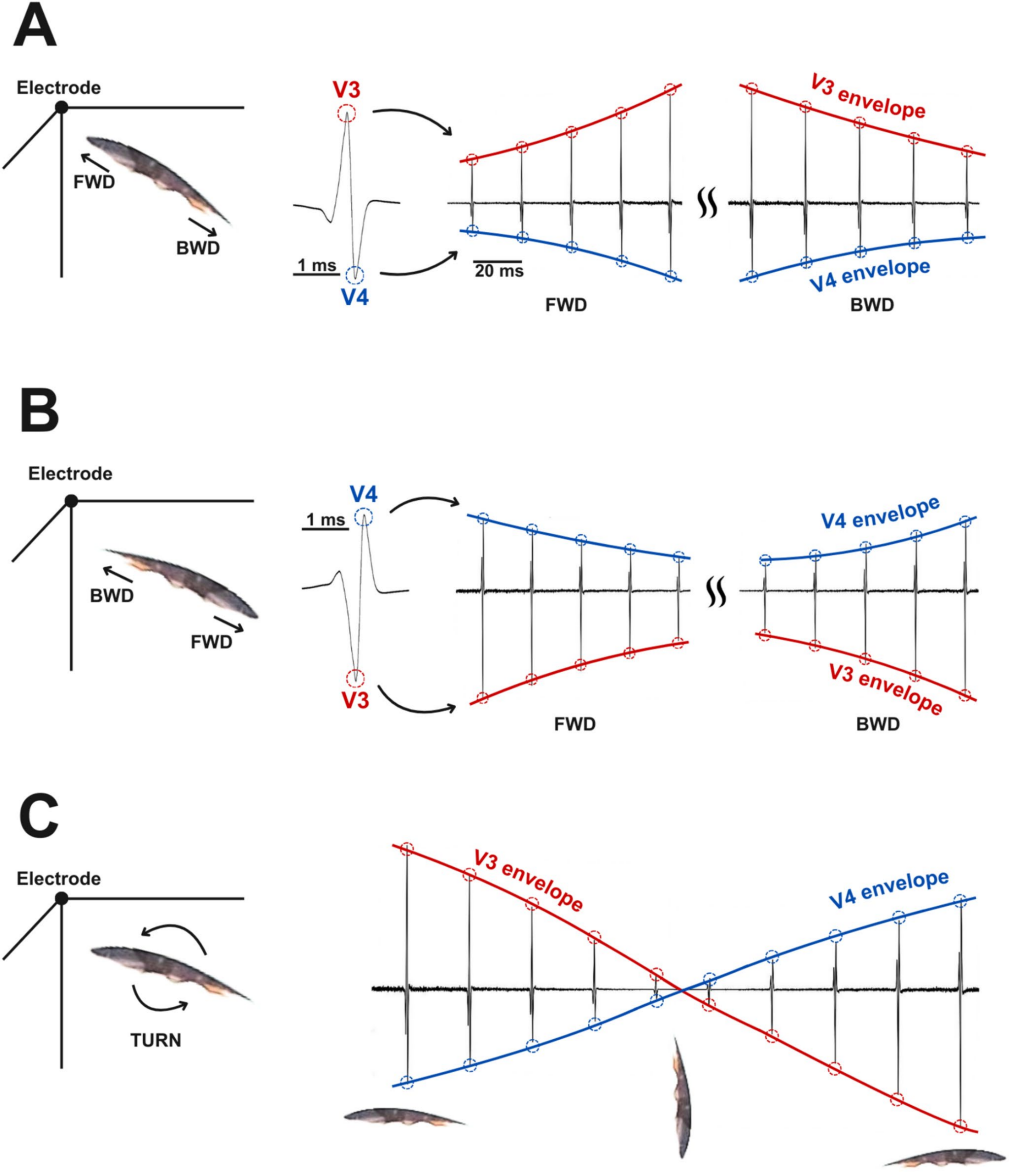
Modulation of the electric signal: V3 and V4 wave components measured in an electrode by fish orientation and proximity. **(A)** Head-oriented position: V3 (red circle) was positive and V4 (blue circle) was negative. Peak amplitudes increased as the fish moved forward (FWD) and decreased as it moved backward (BWD). Connecting the peaks of successive pulses generated smooth envelopes (V3 envelope in red and V4 envelope in blue) that encoded the modulation induced by fish displacement. **(B)** Tail-oriented position: V3 was negative and V4 was positive. Peak amplitudes decreased as the fish moved forward and increased as it moved backward. **(C)** Orientation change: during a turn, V3 and V4 peaks swapped polarities as their envelopes crossed the zero-amplitude axis.

In the time series obtained from a specific electrode, the amplitudes of the V3 and V4 peaks were dynamically modulated by the fish’s movements (Figure 3). Since the V3-V4 sequence was consistently present in all recorded pulses, it was possible, at every single pulse, to identify whether the head or the tail was closer to a given electrode. For instance, if the head was aligned closer to the electrode, the V3 peak at that electrode was positive and the V4 peak was negative (Figure 3A; V3 in read and V4 in blue). On the other hand, if the tail was closer to the electrode, these polarities were reversed (Figure 3B). Consequently, longitudinal movements (forward or backward) were reflected in smooth changes of the peak amplitudes, while a change in orientation (fish turn) caused the peaks to switch polarities (Figure 3C). Given that the fish fired at approximately 40 pulses/s—a rate significantly higher than its displacement speed—a continuity criterion could be applied. This criterion was used to define V3 and V4 envelopes by connecting the peaks of successive pulses. These envelopes effectively captured the amplitude modulation of the stereotyped pulse induced by the fish’s movement.

To measure the experimental V3 and V4 envelopes for each electrode time series, we sampled the peak amplitudes of these components for every pulse of the fish’s electric signal (Figures 3A,B). Initially, a custom GNU Octave script (v. 6.4.0^2^) was used to extract pulse timestamps by calculating the sum of squares of the linearized data across all electrodes, and using a threshold-crossing criterion. This was used for onset detection. For each detected pulse, the maximum positive and negative peaks were identified and organized into envelopes based on their temporal sequence. Subsequently, the envelopes were resampled at 1/30 s intervals to align with the timing of the video recordings; if more than one peak value occurred within an interval, the values were averaged. These experimental data provided a basis for quantitative comparison with simulated envelopes derived from fish-tracking data and spatial charge distribution models of the electric organ’s V3 and V4 peak components.

### 2.5 3D kinematic reconstruction and fish pose estimation

Briefly, the tracking workflow (see Supplementary Figures S1) consisted of training DeepLabCut (DLC v. 3.0; Mathis et al., 2018) to detect 2D skeletal keypoints from a set of fish images and tracking them across all recorded views. These 2D detections were subsequently triangulated to reconstruct 3D kinematic coordinates using Anipose (v. 0.81; Karashchuk et al., 2021). We used the Anipose library with a three-camera setup to increase positional accuracy, resolve pose ambiguities (occlusions), and provide redundancy in data collection. These factors are crucial for the robust analysis of complex animal behaviors.

#### 2.5.1 Training with DLC

To implement markerless pose estimation via DLC using a PyTorch backend, a ResNet-50 architecture (pretrained on ImageNet) was fine-tuned through transfer learning to track three anatomical landmarks: the head, mid-body, and tail tip. These points define a two-segment simplified skeleton representing the fish. The fine-tuning of these deep neural networks was performed on a relatively small set of labeled frames, allowing the model to learn and predict the positions of the chosen fish body parts in new, unseen video footage. To ensure tracking robustness against occlusions and variable lighting conditions, an iterative active learning refinement process was employed. Outlier frames with low-confidence predictions were manually corrected and reintegrated into the dataset. The fine-tuning dataset comprised 2,375 labeled frames extracted from 95 experimental videos of different individuals swimming freely, split into 95% for training and 5% for validation. The model was trained for 80 epochs using a batch size of 22. Performance evaluation (shuffle 3) yielded a filtered root mean square error (RMSE) of 4.59 pixels for the test set and 4.05 pixels for the training set, after applying a confidence threshold (p-cutoff) of 0.7.

#### 2.5.2 Video synchronization, preprocessing, and 2D pose estimation

Following DLC model training, video recording was initiated across the three cameras via a single command; however, a temporal jitter of dozens of frames was observed due to USB protocol overhead. To resolve this, we utilized the first frame in which a blue synchronization LED illumination appeared in each view to trim the sequences and establish a consistent temporal reference. This procedure generated precisely synchronized MP4 videos (recorded concurrently with electric signals, as previously described), which were subsequently processed via DLC to detect 2D skeletal keypoints across all camera frames.

#### 2.5.3 Calibration and 3D reconstruction

Intrinsic and extrinsic camera parameters were determined using the OpenCV calibration tool with a ChArUco board (11 × 8 squares, 297 × 210 mm; Karashchuk et al., 2021) submerged in the experimental tank. This procedure resulted in a final mean reprojection error of 0.75 pixels (~0.3 mm at 960p resolution). To ensure temporal and anatomical consistency, 2D detections were processed using the Viterbi algorithm (as implemented in Anipose) to determine the globally optimal pose sequence. During triangulation, geometric body segment constraints were imposed to reflect the biomechanics of *Gymnotus carapo*. Rigid constraints were applied to the anterior segment (head-to-mid), while soft constraints were assigned to the posterior segment (mid-to-tail) to accommodate the axial flexibility required for undulatory locomotion. Trajectories were further refined using spatial and temporal regularization (smoothing parameters set to 6) and a median filter with a 7-frame window to attenuate high-frequency jitter. This resulted in a frame-by-frame sequence of 3D coordinates referenced to the center of the Camera 1 sensor (Figure 1C).

#### 2.5.4 Spatial alignment and output

For final spatial alignment, the coordinates of the extremities of three reference segments, originating orthogonally from the “REF” electrode position (Figure 1A), were mapped within the reconstruction environment. These reference points were utilized in a custom Python script to compute a change-of-basis matrix, incorporating both rotation and translation. This transformation was applied to the trajectories to convert the Anipose native coordinates into the experimental metric reference frame (defined by the X, Y, and Z axes in Figure 1A). The final output captured the fish skeleton dynamics: a .csv file containing time-resolved 3D coordinates for the head, mid-body, and tail tip relative to the experimental fish tank coordinate system, suitable for subsequent biophysical simulations.

#### 2.5.5 Tracking data precision

To quantify the precision of the integrated tracking pipeline, the three reference segments were recorded across all camera views and processed using the same parameters as the experimental fish videos. The variability of the resulting 3D coordinates was analyzed to assess the system’s spatial reconstruction accuracy. The average standard deviation of the reference segment positions was 0.5 mm. By combining this value with the 0.3 mm error derived from the ChArUco calibration, we obtain a conservative estimate for the total variability of approximately 0.8 mm for each tracking coordinate.

#### 2.5.6 Tracking validation

Anipose provided an output video for manual validation of the tracking. It consists of a frame-by-frame composition with the identified fish skeleton superimposed on the fish images from all three cameras. The video was examined frame-by-frame and all frames containing mislocalizations were identified and discarded from the subsequent model fitting and analysis.

It is important to distinguish occasional mislocalizations from the system’s baseline reconstruction accuracy of 0.8 mm per coordinate. While the latter represents the inherent precision of the Anipose 3D tracking, mislocalizations occur when DLC fails to correctly identify or label a skeletal point in a specific 2D camera frame. Consequently, the 3D position may be miscalculated due to incorrectly marked 2D inputs, even though the system’s underlying triangulation accuracy remains constant throughout the analysis.

### 2.6 Simulation of the envelopes from the video tracking skeleton dynamics

**Figure 4:**
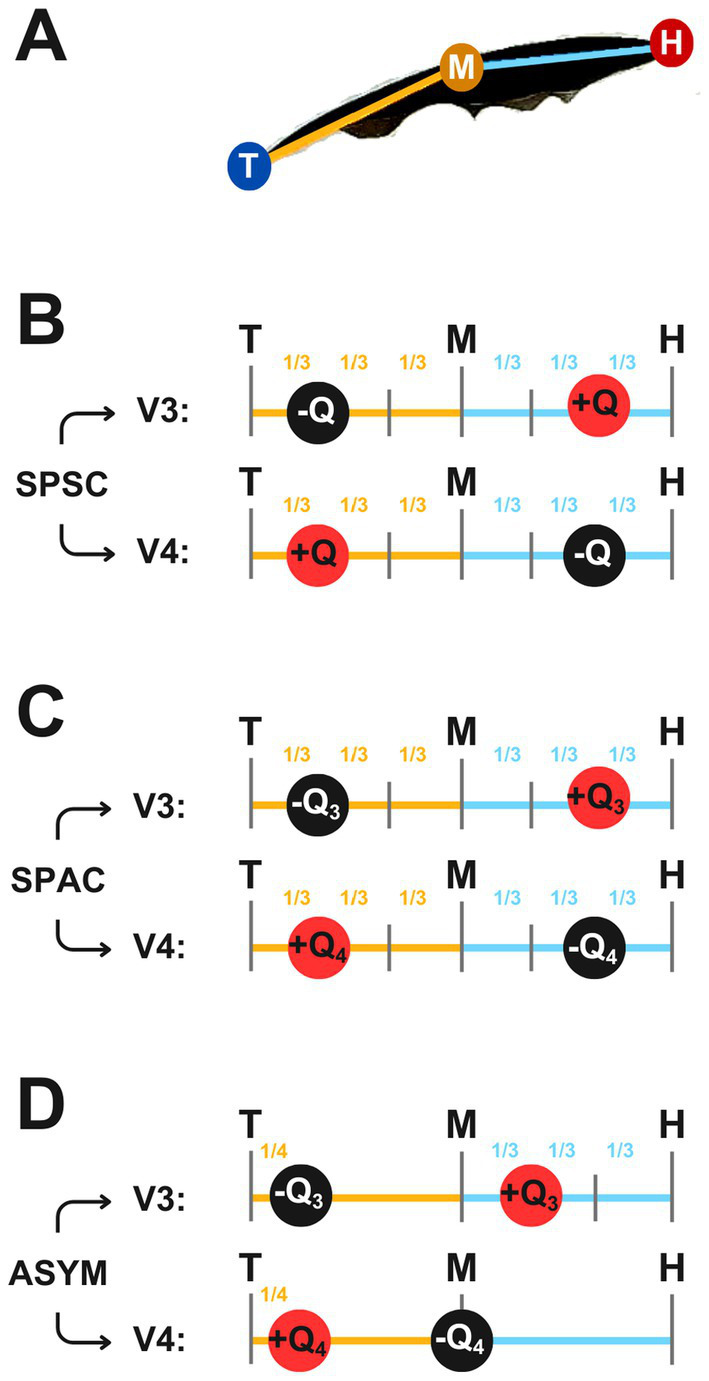
Detected fish skeleton and geometric models of spatial charge distribution along the electric organ. **(A)** Two-segment fish skeleton superimposed onto the fish image. The anatomical keypoints—head (H), mid-body (M), and tail tip (T)—defined the segments where point charges were positioned according to each model. **(B)** Dipole-inspired SPSC (symmetric in position and symmetric in charge) model; envelopes were simulated using a constant charge magnitude Q, with polarities swapped when transitioning from V3 to V4. **(C)** SPAC (symmetric in position and asymmetric in charge) model; maintained the same spatial symmetry as SPSC, but the charge magnitudes Q3 and Q4 were allowed to vary independently for V3 and V4 simulations. **(D)** ASYM (asymmetric) model with biologically inspired geometry; the anterior charge shifted position between V3 and V4, while the posterior charge remained fixed. In all models, positive and negative charges were represented in red and black, respectively. Simulations were initially performed with a 1 μC charge magnitude, which served as a baseline; this value was subsequently used as a scaling parameter to fit the simulated envelope amplitudes to the experimental data.

A typical simplified skeleton detected by video tracking is shown in Figure 4A, superimposed onto the fish image from one of the camera views, with three anatomical keypoints identified: head (H), mid-body (M), and tail tip (T). Simulating the electrical potentials at the electrode array requires defining both the fish position and pose relative to the array (method described above). It also requires a spatiotemporal charge distribution model for the electric organ, which spans the length of the fish (Bennett and Grundfest, 1959).

For simplicity, we assumed that the temporal evolution of the pulse pattern can be modeled as an oscillation comprising only two components: the V3 peak followed by the V4 peak. Under this simplification, calculating the potential difference between a given electrode and the reference electrode for each frame yielded simulated V3 and V4 envelopes. These were then quantitatively compared to the experimental envelopes described previously. Furthermore, instead of using a continuous spatial distribution, we approximated the EO as two discrete effective sources (modeled as point charges) of opposite polarity. These charges were positioned along the simplified skeleton segments. The magnitudes of the positive and negative charges were equal, ensuring the electroneutrality of the electric organ at all times.

The geometry of the **Symmetric in Position and Symmetric in Charge (SPSC)** model, (Figure 4B), was based on the classical symmetric dipole model. This model is traditionally used as a first approximation for the EO (Bullock et al., 2005) or for stimulating live fish with a pair of wires driven by artificially generated symmetric pulses (Jun et al., 2013; Lareo et al., 2016). In this model, both V3 and V4 components are simulated by two charges (+Q and –Q) situated at one-third of the distance from the segment endings; the charge polarities are then inverted when transitioning from V3 to V4. Unlike a standard static dipole, this model accounts for the fish’s flexibility. Because the skeleton segments can rotate at the mid-body (M) junction, the spatial distance between the charges is not constant; instead, it is a function of the fish’s posture. This allowed the simulation to capture how body bending influenced the resulting electric field and envelopes.

The **Symmetric in Position and Asymmetric in Charge (SPAC)** model (Figure 4C) differed from the SPSC in that the charge magnitudes varied between the two components, specifically denoted as ±Q3 and ±Q4. This configuration is analogous to the two-wire solution employed in lab interactions with live electric fish, wherein previously recorded asymmetric electric pulses are reproduced (Waddell and Caputi, 2021).

Lastly, the **ASYM (Asymmetric in Position and Asymmetric in Charge)** model (Figure 4D) introduced spatial asymmetry in the charge distribution. The ASYM provides a more biologically realistic representation of the fish’s EOD, because unlike the previous models, the ASYM geometry was inspired by experimental data on local electric field gradients and current flows (Caputi and Budelli, 1995; Figure 5.4 in Caputi, 2025), Simulated envelopes were first generated using a 1μC charge magnitude across all models. These were then fitted to the experimental envelopes by varying the charge magnitude as a free parameter to minimize the amplitude difference between the waveforms.

The envelope simulations were based on six water-glass/air interfaces, forming a virtual tank with the same dimensions as the experimental setup. We implemented this environment using a custom Python program (code available at https://github.com/Rafelseixas/Gymnotus-carapo-simulation) that calculates the electric potential at the coordinates of the physical electrodes, placed at the eight corners. The program retrieved the skeleton’s position and pose dynamics from the tracking-derived .csv files. It computed the coordinates of all charges within the virtual tank for each frame based on the selected geometric model. Since the frequencies involved in the pulse shaping are sufficiently low, we could adopt a quasi-static approximation, under which electric fields can be treated as instantaneously established.

We modeled the simulation as an electrostatic problem, calculating the electric potential frame-by-frame via Coulomb’s Law and the superposition principle. The electrostatic representation was chosen over current sources (Lissmann and Machin, 1958) due to the ease of applying electric field continuity at the interfaces to account for media polarization effects in the simulations. To satisfy the boundary conditions at the dielectric interfaces, the method of image charges was employed (Jeans, 1960; Smythe, 1968; Jackson, 1999; Griffiths, 2013; Lissmann and Machin, 1958). The electric field generated by a point charge Q located within a dielectric medium *ϵ*1​, near an interface with a second dielectric ϵ2​, polarized the molecules of both media. As the polarization was more pronounced in the medium with higher permittivity, a net bound surface charge was induced at the interface. The method of images simplified the problem by invoking the uniqueness theorem, replacing the complex continuous surface charge distribution with a single virtual point charge Q′. This virtual charge was placed at the mirror-image position within medium 2 such that the resulting potential satisfied the boundary conditions at the interface:


$$Q^{\prime }=\beta Q.$$ (2)

β=(ϵ1−ϵ2)/(ϵ1+ϵ2), (3)

where Q is the real point charge, Q′ is the first-order image charge, β is the reflection coefficient, and ϵ is the permittivity of the dielectric medium. In a fully enclosed rectangular domain, such as the water-filled glass tank, a single charge would require six first-order images Q′ - one for each interface.

However, the surface distributions induced at each face interacted at the 12 tank edges, necessitating second-order image charges:


$$Q^{\prime \prime }=\beta^{2}Q.$$ (4)

Furthermore, the interactions at the intersection of these edges induced charges at the eight corners, requiring third-order images:


$$Q^{\prime \prime \prime }=\beta^{3}Q.$$ (5)

While parallel faces theoretically generated an infinite sequence of reflections, we truncated the expansion at the third order. This approach accounts for the critical orthogonal coupling across the tank walls. It was particularly effective for sensors located near the corners, where the influence of multiple wall intersections was most significant. Consequently, for each real charge in the water, we also computed the cumulative effect of 26 image charges (6 first-order + 12 s-order + 8 third-order).

The decision to truncate the image series at the third order was quantitatively justified, even for high water-glass reflection coefficients (β = 0.85). At the tank vertices, where boundary effects were most pronounced, the potential was dominated by the local image cluster (Orders 1–3). The error introduced by neglecting fourth-order reflections –which represented distant wall interaction– was estimated to be less than 1.5% for source charges located within 10% of the edge length from the corner. This error margin was significantly lower than the typical noise floor encountered in electrophysiology, electronics and pose-tracking.

Following the computation of the absolute potentials at each vertex, we determined the differential potential at the position of each physical electrode in the array (“X,” “Y,” “Z,” etc.) relative to the designated reference point. This subtraction process yielded the simulated values for V3​ or V4​ (depending on the specific charge distribution being modeled), replicating the differential measurement configuration and lead topology of our experimental data acquisition system in a frame-by-frame time sequence. These simulated envelopes served as the basis for a parameter optimization procedure. Charge magnitudes were tuned to match the peak amplitudes of the experimental V3​ and V4​ signals. This calibration ensured that the simulated models are compatible with the bioelectric output of the subject.

In addition to simulating V3​ and V4​ from the skeleton-based dynamics, we implemented a secondary version of the simulation to evaluate the V3​–V4​ asymmetry for each specific charge geometry model. In this configuration, the subject was represented by aligned segments positioned at the geometric center of the virtual tank. Electric potential was sampled at points located at a fixed distance of 1 cm from the head and tail. This setup allowed for the simulation of the classical longitudinal head-to-tail pulse pattern (Figure 2A), providing a controlled baseline to validate the model’s sensitivity to axial charge distributions.

### 2.7 Results validation

For results derived from video-tracking, we performed a direct temporal comparison between simulated and experimental V3​ and V4​ envelopes across all electrodes, calculating the cumulative sum of squared errors (SSE) on a frame-by-frame basis.

To assess the distributional similarity independent of time, we utilized Quantile-Quantile (Q-Q) plots, which compared the probability distributions of simulated and experimental values (Wilk and Gnanadesikan, 1968), previously used to quantify EOD distributions (Forlim et al., 2015). To quantify the agreement between these distributions, we calculate the Q-Q plot average model mismatch, a modification of the Tukey mean-difference analysis (Forlim et al., 2015). To that end, we measured the area under the quantile-quantile curves and took the average. This method allowed us to identify systematic biases and limits of agreement between the simulated model and the experimental data.

The different models were compared using the V3​–V4​ asymmetry ratio. Since this metric was independent of tracking data and depends solely on the geometric charge distribution along the subject’s body, it served as a robust indicator of the model’s structural validity.

## 3 Results and discussion

To demonstrate the proposed methodology, we present a comparative fit analysis of a representative 65-s dataset consisting of synchronized video and electric signal recordings from one 16-cm freely swimming *Gymnotus carapo*. This example serves to illustrate the workflow, which remains equally effective and scalable for future extended recording durations for pulse-type Gymnotus.

### 3.1 Experimental envelope extraction and fish orientation analysis

The analysis of the electrical recordings began by importing the Axon .abf files into Octave. Since every electrode in the array is referenced to the same REF electrode (see coordinate system in Figure 1A), we will henceforth refer to the signal as that of electrode *i* (*i* = “X,” “Y,” “Z,” “XY,” “XZ,” “YZ,” or “XYZ”). Each electrode time series is approximately 70 s long and contains about 3.5 million data points recorded at 50 kS/s. No signal saturation was observed at any electrode across the entire dataset. Recorded values remained strictly between −9 V and +9 V, well within the ±10 V saturation limits of the system. The inverse gain curve of the corresponding differential amplifier was applied to the data from each electrode to reconstruct the original amplitude of the fish-induced signals. Following this, all EOD pulses were detected, and their timestamps were extracted. For every pulse, the V3​ and V4​ peaks were identified, and the envelope data were resampled to match the video frame rate (30 fps, 1/30 s). A total of 3,545 pulses were detected in this dataset, representing an average discharge rate of 50.6 pulses/s.

#### 3.1.1 Experimental envelope extraction

**Figure 5:**
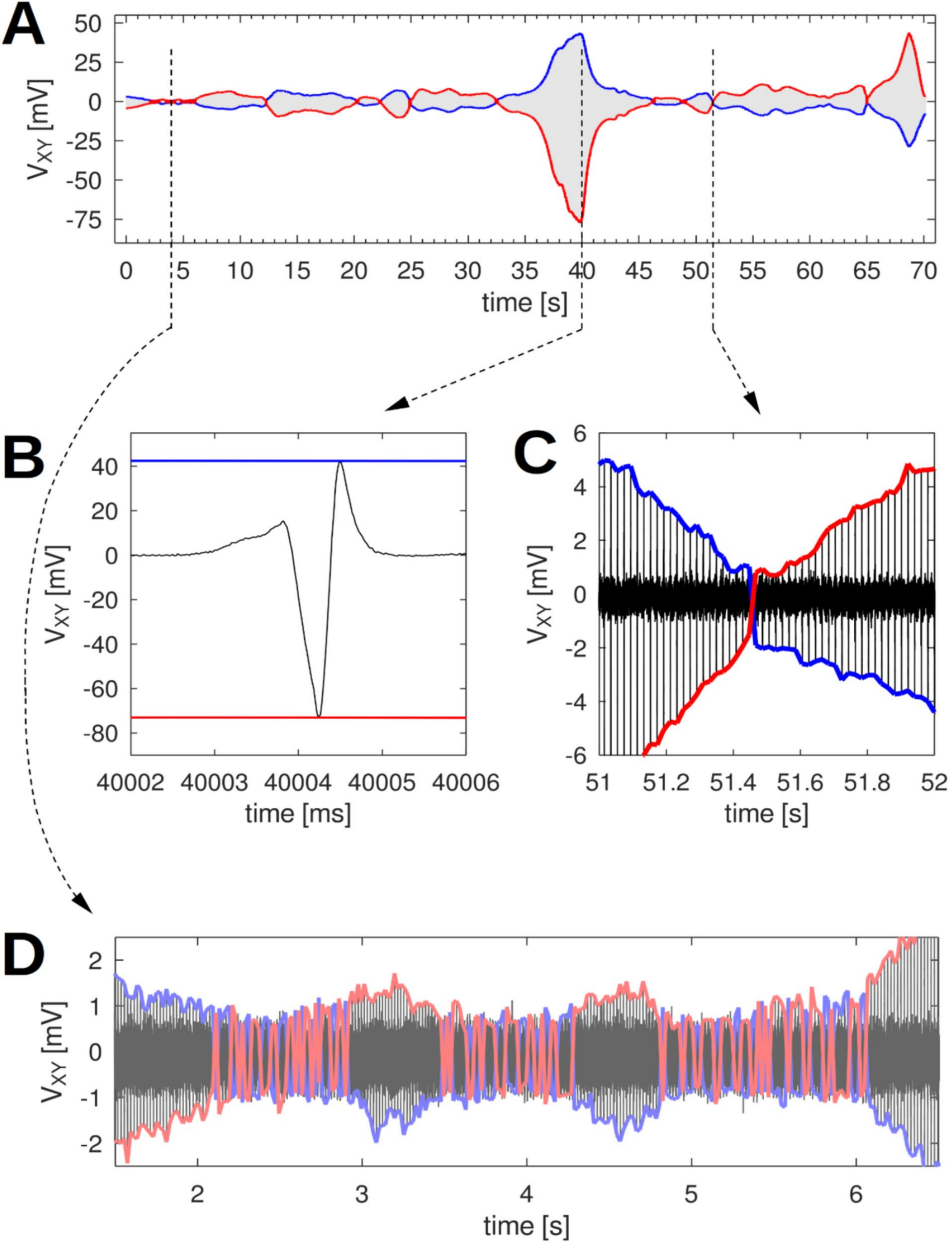
Representative experimental time-series with V3 and V4 envelopes. **(A)** Electrode “XY”: Raw time-series (light gray) shown with V3 (red) and V4 (blue) envelopes. The 70 s recording comprises approximately 3.5 million data points acquired at 50 kHz (3,545 EODs); the high density of data points results in the solid gray appearance between envelopes. Note the multiple polarity reversals as envelopes cross, with a maximum amplitude difference of ~120 mV occurring near *t* = 40 s. Dashed vertical lines mark regions of interest detailed in subsequent panels. **(B)** Magnified view at *t* = 40 s, showing a single ~120 mV EOD pulse with a negative V3 peak and a positive V4 peak. **(C)** Detail of V3 and V4 envelope crossing at *t* = 51.5 s. The EODs exhibit dynamic amplitude changes: initially, V3 is negative and V4 is positive (tail toward the electrode); subsequently, both amplitudes vanish due to fish rotation and swap polarities, ending with V3 positive and V4 negative (head toward the electrode). The darker region near zero amplitude represents the total noise—originating primarily from the signal chain—with a peak-to-peak amplitude of approximately 1 mV. **(D)** Signal behavior around *t* = 4 s, where the signal-to-noise ratio at electrode “XY” is too low (due to the fish’s distance from the electrodes) to allow stable detection of V3 and V4.

Figure 5A shows a typical time series of the reconstructed data (gray) along with the corresponding V3 (red) and V4 (blue) envelopes for electrode “XY.” The area between the envelopes appears solid gray due to the high density of data points (see components V3 and V4 in Figure 2A). Several crossovers between V3 and V4 were observed, indicating that the fish’s alignment relative to electrode “XY” changed throughout the experiment (see Figure 3C).

The maximum pulse amplitude occurred at approximately 40 s (Figure 5B). In this zoomed view, we observe an inverted pulse (negative V3 and positive V4) with an asymmetry where V3 amplitude is approximately twice that of V4. This ratio significantly deviates from the −21% value of the classic tail-to-head pattern, clearly indicating that the fish is at an asymmetric distance from the electrodes.

Figure 5C illustrates a sequence of approximately 50 pulses near *t* = 51.5 s and their corresponding envelopes. On the left of Figure 5C, the V4 envelope is positive and the V3 envelope is negative, indicating an orientation primarily tail-oriented toward the electrode. Subsequently, the pulse amplitudes vanished and the envelopes inverted their polarities; to the right of the figure, the amplitudes increased again, indicating a transition to a head-oriented position. This movement represents the inverse of the turn previously illustrated in Figure 3C.

Additionally, the amplitude of the dense central region along the time axis indicates a total noise of approximately 1 mV. This measurement reflects primarily the electromagnetic pick-up and electronic noise accumulated along the entire signal chain—which cannot be completely shielded by the Faraday cage due to the camera system—far exceeding the baseline noise in the water.

Finally, Figure 5D (a magnified view around *t* = 4 s) illustrates the effects of this noise on envelope determination when the fish is far from the electrode “XY.” On the left of Figure 5D, the signal behavior mirrors that of Figure 5C, indicating a primarily tail-oriented position followed by a decrease in pulse amplitude. Although an envelope reversal is visible at *t* = 2.5 s, the low signal-to-noise ratio causes successive oscillations in the detected envelopes. These envelope oscillations eventually stabilize for *t* > 6.0 s, once the signal amplitude increases sufficiently to overcome the noise.

While total noise can reach 1 mV, the signal-to-noise ratio is only problematic in extreme cases, such as when the fish is distant from or oriented away from an electrode (Figures 5C,D). However, the electrode array’s spatial distribution guarantees that these conditions never simultaneously affect all sensors.

#### 3.1.2 Multi-electrode envelope comparison

**Figure 6:**
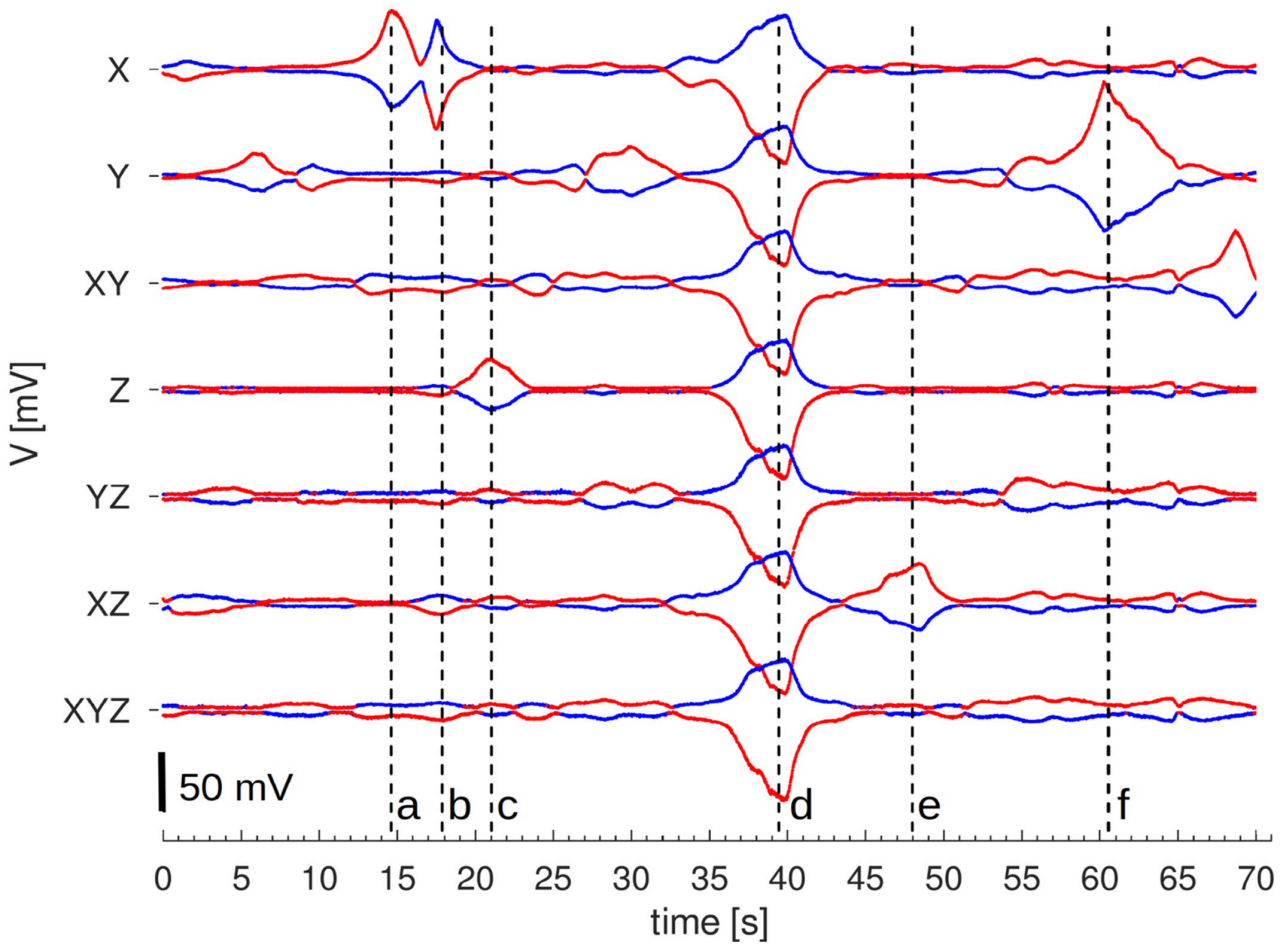
Experimental V3 (red) and V4 (blue) envelopes obtained for all electrodes in the matrix. All vertical scales are identical, as indicated by the 50 mV reference bar in the bottom left. Vertical dashed lines labeled (a–f) represent specific spatial orientations of the fish in the tank that will be analyzed in further detail. To provide more clarity for lower amplitudes, signals from different electrodes were partially superimposed around *t* = 40 s, where a highly consistent behavior was observed across all electrodes.

The envelopes obtained for all electrodes in the array during the experiment are shown in Figure 6, plotted on the same amplitude scale. Dynamic amplitudes and envelope crossovers are observed across all electrodes. Notably, at any given time, there is always at least one electrode where the pulse amplitude is significantly higher than the noise floor. This redundancy validates the effectiveness of the electrode array in reliably detecting the electric organ pulses, regardless of the animal’s position or orientation within the tank, as we already reported before (Forlim and Pinto, 2014; Matias et al., 2015; Guariento et al., 2016). Vertical dashed lines were positioned at six representative time points (a–f) throughout the experiment to illustrate how orientation and positional information are dynamically encoded in the electrical recordings.

#### 3.1.3 Correspondence with video recordings

**Figure 7:**
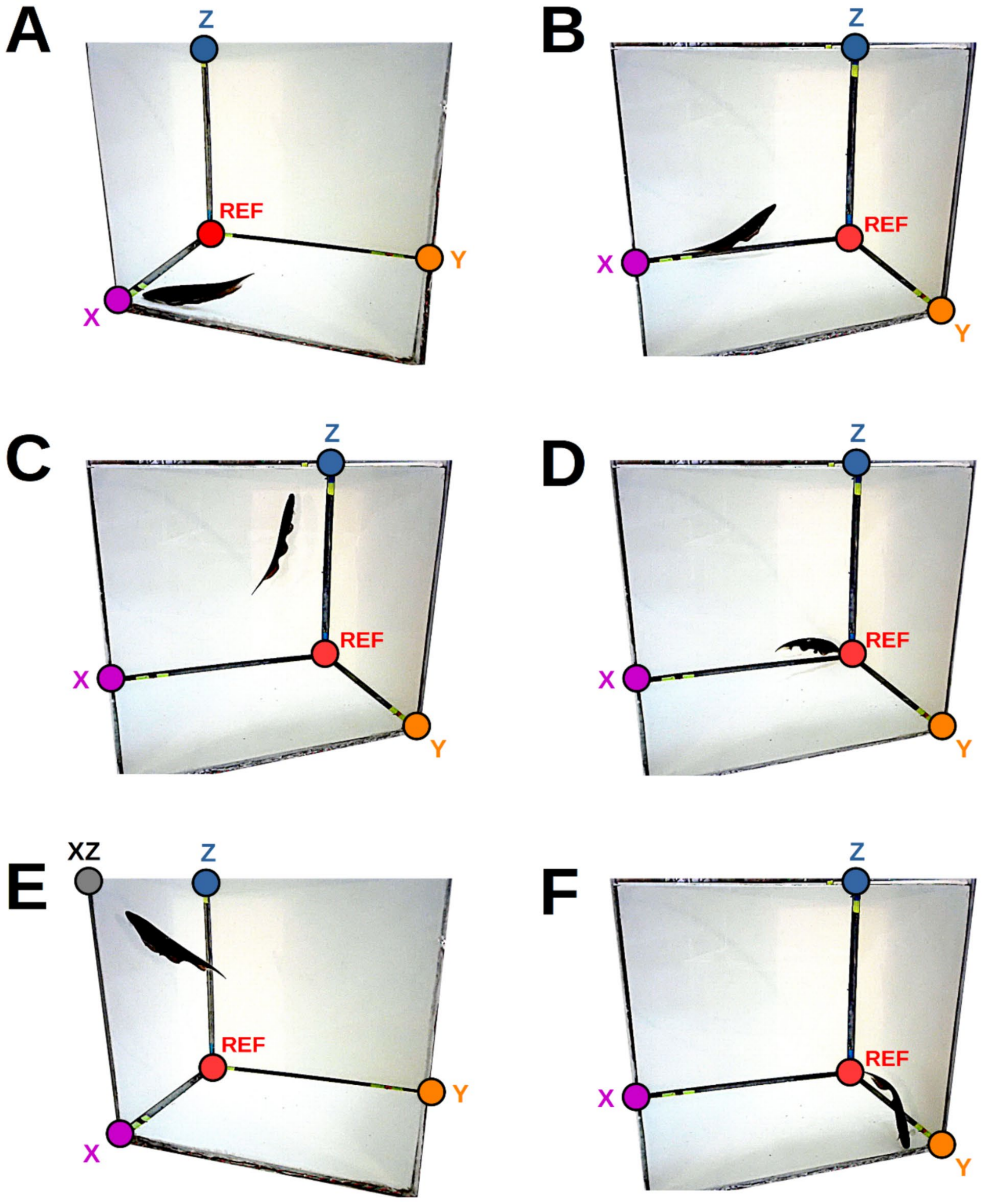
Examples of fish displacement throughout the experiment. **(A–F)** Correspond to the details marked (a–f) in Figure 6. Frames **(A)** and **(E)** were recorded with Camera 1; all others were captured with Camera 2. The contrast of the original images was increased, and the positions of some electrodes from the array were superimposed (colored circles; same color code as in Figure 1) as a spatial reference. The position of electrode “XZ” was also added to panel **(E)** for clarity. All electrodes within the array were present in the frames, but some were omitted from the panels to avoid visual clutter.

Figure 7 presents a series of frames from the video recordings (see Supplementary Video S1) to illustrate the fish’s position and orientation at the specific time points selected from the electrical data in Figure 6. Specifically, Figures 7A,E are frames extracted from the Camera 1 feed, while Figures 7B–D,F were obtained from Camera 2. We combined images from two different cameras to provide the optimal 2D perspective for illustrating the fish’s 3D position and orientation at each specific chosen moment.

**Figure 8:**
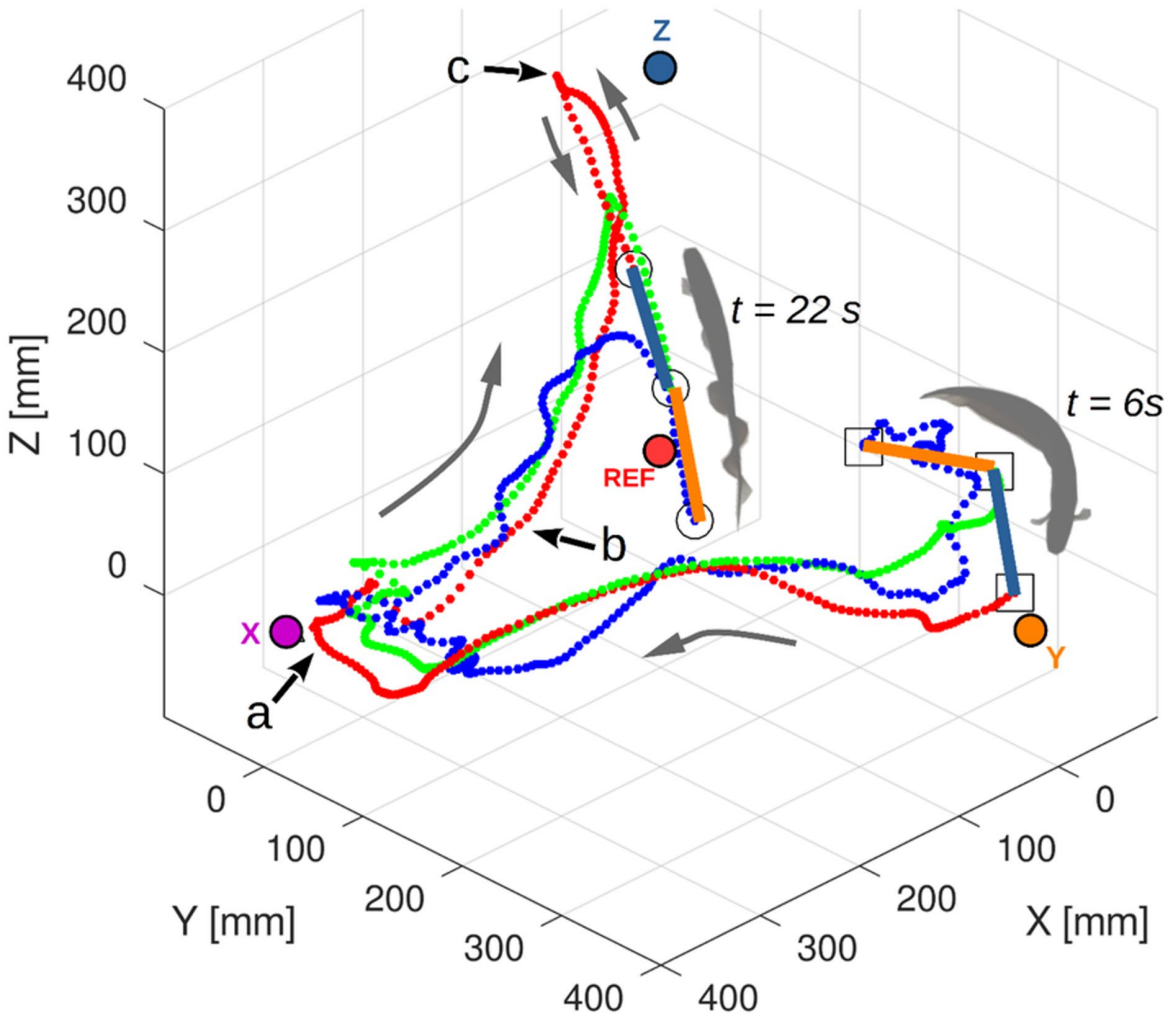
Excerpt of a 3D tracking skeleton trajectory. The 3D plot perspective is positioned between those of Cameras 1 and 2. Electrodes “REF,” “X,” “Y,” and “Z” are superimposed on the plot for reference. Coordinates correspond to the actual dimensions of the experimental tank. Trajectories of the key skeleton points—head, mid-body, and tail—are represented by red, green, and blue dots, respectively (see anatomical keypoints T, M, and H in Figure 4A). Skeleton segments for head-to-mid-body (blue) and mid-body-to-tail (orange) are shown at the start (squares) and end (circles) of the displayed interval, with adjacent low-opacity images illustrating the fish’s orientation at those moments. Gray arrows indicate the direction of movement. Head positions along the red trajectory, pointed a, b, and c, correspond to the timepoints marked in Figure 6 (a–c) and **(A–C)** in Figure 7. The plotted trajectories illustrate a portion of free exploratory behavior, beginning at *t* = 6 s with the fish positioned near electrode Y, progressing past electrode X, turning towards electrode “Z,” and concluding at *t* = 22 s as the fish swims backward from the proximity of electrode “Z.”

Figure 7A was captured at *t* ≈ 14.5 s and Figure 7B shortly thereafter near *t* = 18 s. In both frames, the fish was closest to electrode “X,” which accounts for the higher pulse amplitudes recorded at this electrode compared to the others (Figure 6 dashed lines a and b). A clear change in the fish’s orientation relative to electrode X is visible between Figure 7A (head toward electrode) and Figure 7B (tail toward electrode), resulting in the polarity reversal of V3 and V4 (Figure 6). Figure 7C shows the fish near electrode “Z” with its head oriented toward it at *t* ≈ 20 s. This proximity and alignment result in both the highest amplitudes at this electrode compared to the others and the specific positive V3 / negative V4 polarity (Figure 6 —dashed line c).

Figure 7D shows the fish with its head near the “REF” electrode at *t* ≈ 40 s, explaining why all electrodes in Figure 6 (dashed line d) simultaneously exhibit maximum signal amplitudes. This also accounts for the inverted pulse shown in Figure 5B: since the head is oriented toward the common reference, all electrodes capture a pattern mirroring the classic tail-to-head signal but with reversed polarity. Furthermore, the proximity to the reference point explains the distinct V3–V4 asymmetry observed during this interval.

Finally, in Figures 7E,F, the fish approaches electrodes “XZ” and “Y” with its head, respectively, producing effects in the electrical data (Figure 6 —dashed lines e and f) similar to those observed during the approach to electrode “Z.” Notably, the fish’s body being more closely aligned with the “Y”– “REF” axis in Figure 7F results in the larger pulse amplitude of electrode “Y” observed at dashed line f, further demonstrating how the signal magnitude depends on the projection of the EOD onto the electrode’s recording axis.

To compare these results from a current source perspective for the EO, we used the relation:


I=4πσrV, (6)

where I is the current source intensity, σ is the water conductivity (100 μS/cm), r is the distance between the source and the electrode, and V is the electric potential generated (Lissmann and Machin, 1958). Based on the data in Figure 6 (line d) and Figure 7D, with V = 77 mV (peak of V3) and r ≈ 1 cm (head-to-electrode distance), we estimated the peak current of the OE to be I ≈ 1 mA. This value is consistent with those reported in the literature (e.g., Caputi and Budelli, 1995).

### 3.2 3D tracking and its correlation with EOD envelope dynamics

Following data acquisition with free-swimming fish, video footage from three cameras was synchronized with electrical recordings and processed as detailed in section 1.5. The resulting dataset stored in a .csv file comprised the anatomical coordinates for the head, mid-body, and tail for 1,936 frames (∼64.5 s). A validation video overlaying the identified skeleton onto the raw footage was also generated (see Supplementary Video S1).

Figure 8 displays a representative 3D skeleton trajectory. Trajectories for the head, mid-body, and tail are plotted at two-frame intervals in red, green, and blue, respectively, capturing a portion of the fish’s freely exploratory behavior. Since the recording frame rate is constant (30 FPS), the higher point density observed along certain sections of the trajectory denotes a lower swimming velocity. Notably the head trajectory is significantly smoother than that of the tail, which exhibits several oscillations during the interval, as is also evidenced in Supplementary Video S1. The trajectories begin at frame 180 (*t* = 6 s), with the fish near electrode “Y” (skeleton segments marked by squares). The fish swims diagonally toward electrode “X,” turns, and approaches electrode “Z” near the surface. Subsequently, it rapidly swims backward—a characteristic behavior of this species—and the trajectories end at t = 22 s (skeleton segments marked by circles). Labels a, b, and c denote head positions corresponding to the same specific timepoints in Figures 6, 7A–C. These dynamics are further confirmed in Supplementary Video S1.

Remarkably, the initial proximity of the head to electrode ‘Y’ coincides with the maximum envelope amplitudes and the specific polarity observed in Figure 6 (*t* ≈ 6 s). As the fish approaches electrode ‘X’, a corresponding amplitude increase appears near *t* = 15 s (detail a). The subsequent turn towards electrode ‘Z’ correlates precisely with the polarity reversal (Figure 6, a,b). Finally, the fish’s proximity to electrode ‘Z’ aligns with the high amplitude observed in detail c.

These findings support the idea that trajectory information is reflected in the envelope dynamics. The point-to-point coincidence between local maxima, polarity reversals, and kinematic events observed in the 3D tracking validates this correlation. This suggests that the electrical envelopes can be used as a proxy for the fish’s spatial trajectory and orientation.

**Figure 9:**
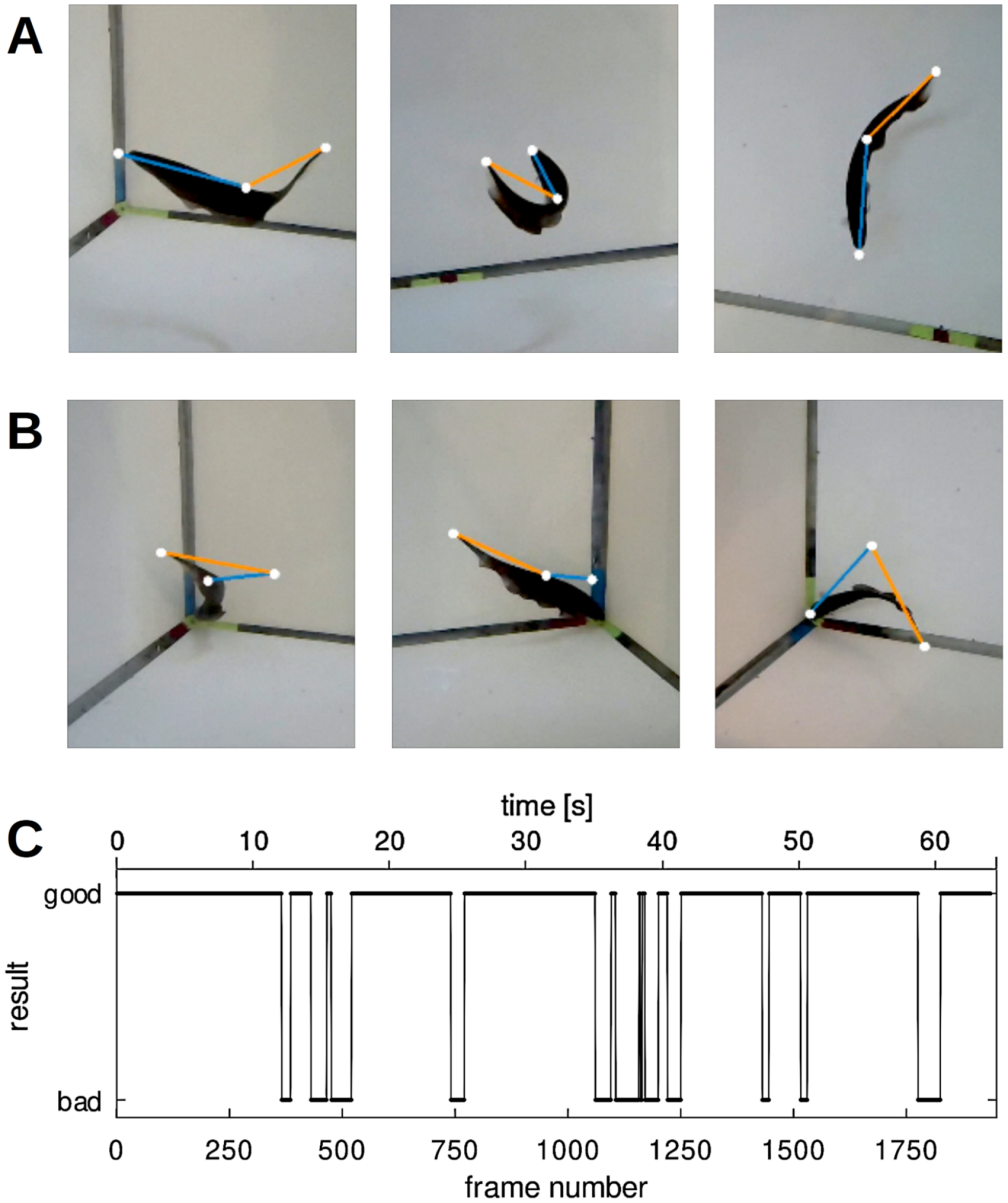
Tracking validation. **(A,B)** Representative images of fish skeleton tracking used for manual validation. Panels show details from Cameras 1 (front), 2 (side), and 3 (top) from left to right (see Figure 1). Skeleton key points are marked with white circles; the head-to-mid-body segment is shown in blue, and the mid-body-to-tail segment in orange. Images were extracted from Supplementary Video S1. **(A)** Details from video frames at *t* = 42.13 s. The three skeleton points are accurately localized in all camera views, despite the body curvature. **(B)** At *t* = 38.0 s, the head is misidentified in the front and side views, while the mid-body point is incorrectly positioned in the front and top views. **(C)** Manual validation performance throughout the experiment. Data points indicate the status of 3D skeleton localization on a per-frame basis. A conservative validation criterion was applied: any frame containing at least one incorrectly positioned key-point in any of the camera views was flagged as invalid. The dual *x*-axis displays both the elapsed time (top) and the absolute frame number (bottom), with videos recorded at a constant frame rate of 30 FPS.

To assess the accuracy of the tracking data, the validation video (Supplementary Video S1) was carefully examined frame-by-frame. Figures 9A,B illustrates two representative examples of this validation at distinct time points. Manual inspection confirmed that key skeleton points were correctly localized across all camera views, successfully validating 1,575 out of 1,936 frames—an accuracy greater than 81%. The temporal distribution of these validated frames throughout the experiment is shown in Figure 9C. All mislocalized frames were excluded from model fitting and analysis.

The 19% of skeleton point mislocalizations were distributed throughout the experiment, occurring primarily when the fish’s projection was captured against the aquarium corners in multiple camera views. In these regions, the convergence of edges created visual artifacts—patterns of color and texture—that we believe the tracking algorithms occasionally confounded with the fish’s anatomy. Examples of these instances occurred near frames 450 (15 s), 750 (25 s), 1,200 (40 s), and 1,800 (60 s). However, these mislocalizations were intermittent; even in the most challenging scenarios, a sufficient number of frames were correctly localized to maintain near-continuous tracking. Notably, several mislocalizations occurred between frames 300 and 550 (10–18 s), within the trajectory shown in Figure 8 (specifically as the fish approached electrode “X”). All frames containing mislocalizations were identified and discarded from the subsequent model fitting and analysis. Furthermore, to mitigate such edge effects in future studies, a redesigned experimental tank is currently under development.

### 3.3 Envelope simulations and charge-distribution model comparison

Envelope simulations were performed using a custom Python script that integrated tracking data to distribute two electric charges, ±Qi (initial values of ±1 μC), across the two fish segments, following the designated charge distribution model (±Q3​ for V3​ and ±Q4​ for V4​). Subsequently, the charge magnitudes were rescaled—while strictly maintaining the electrical neutrality of the EO as a whole—to align the simulated envelope amplitudes with the experimental data, as detailed in the Methods section.

**Figure 10:**
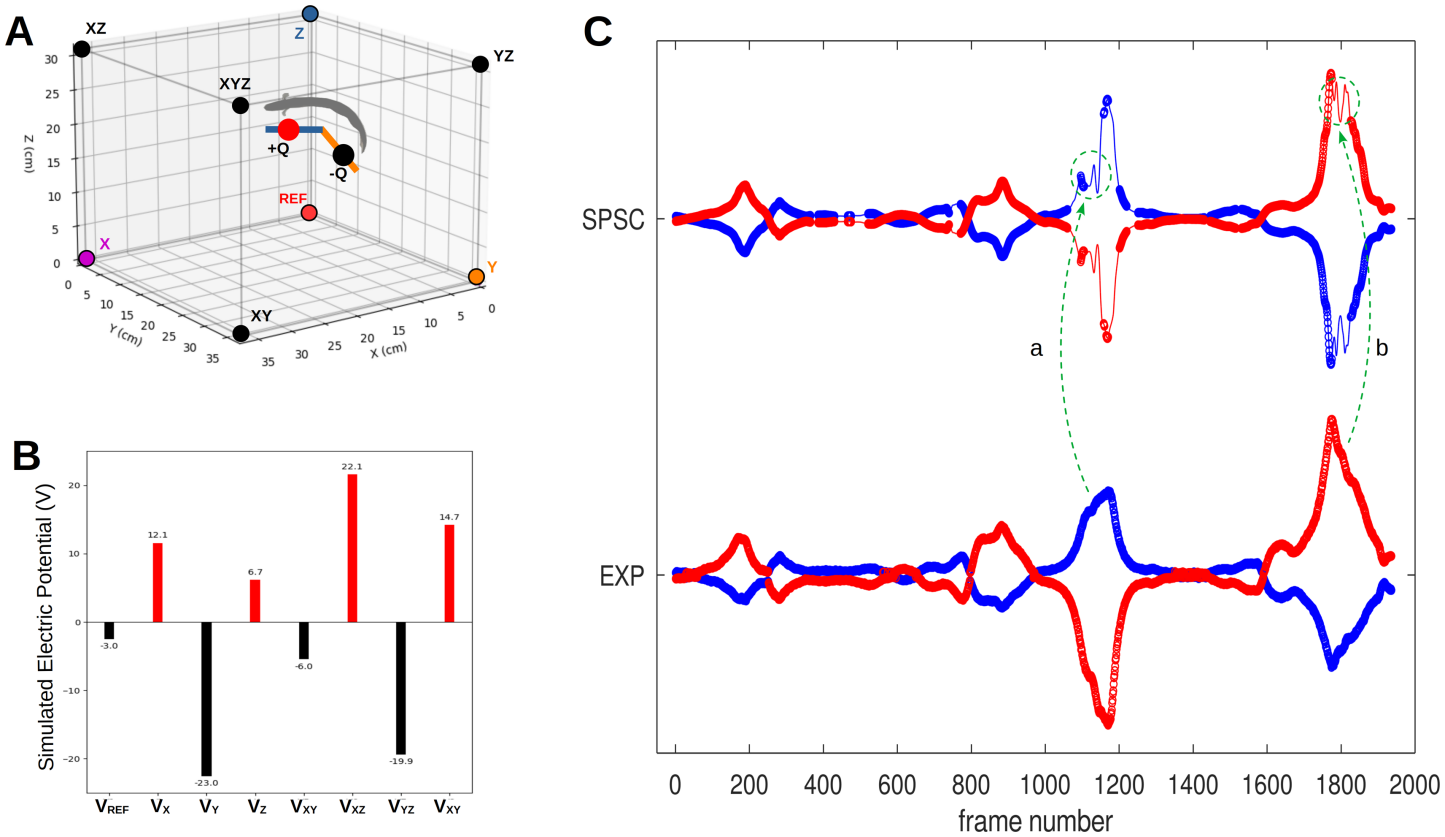
Envelope simulation. **(A)** Fish skeleton derived from tracking data immersed in the virtual tank replicating the experimental geometry. A superimposed semi-transparent image illustrates the actual fish posture, with the 3D plot perspective aligned with the tracking data plots. V3 component (head-segment-positive) source charges (±1 μC) were positioned at one-quarter of the segment length from the extremities. **(B)** Electric potentials at the electrodes are calculated via Coulomb’s law and the superposition principle, accounting for the source charges and their 52 image charges (up to 3rd order). **(C)** SPSC model and experimental V3 and V4 envelopes at electrode Y. (Top) SPSC model: circles correspond to results from validated frames only (Figure 9C), while solid thin lines represent all tracking data. Amplitudes were normalized to a common scale to facilitate qualitative comparison. (Bottom) Experimental envelopes. V3 envelopes are in red and V4 in blue. Green dashed lines (details a and b) indicate small oscillations present in the experimental data that appear magnified in simulations derived from non-validated tracking data.

Figures 10A,B depicts the simulation scenario and the electric potentials (V3​ component of the pulse, head-to-middle-body segment positive) obtained at the electrodes for a representative frame. The fish skeleton coordinates (in cm) were: head (24.7, 25.0, 23.9), middle-body (17.2, 30.4, 21.4), and tail (20.7, 36.8, 15.6). Charges were positioned at one-quarter of the segment length from the extremities. It should be noted that these are hypothetical positions used specifically for this illustrative simulation and remain distinct from the refined spatial parameters evaluated in the SPSC, SPAC, and ASYM models. The program computed the electric potentials at the electrodes generated by the two source charges and their 52 image charges (up to third-order), using Coulomb’s law and the superposition principle. The fish skeleton was positioned within a virtual tank reproducing the geometry of the experimental setup (Figure 10A). The V3​ amplitude at electrode *i* for this frame was calculated by subtracting the electric potential of the reference electrode (V_REF_​) from the potential at the electrode (V_i_​):


$${\mathrm{V3}}_{i}=V_{i}-V_{\mathrm{REF}},$$ (7)

where i is an electrode, V_i_ is the potential at the electrode i and V_REF_​ is the electric potential of the reference electrode.

This simulation procedure was applied to both the V3​ and V4​ pulse components across all tracking frames for the three geometric charge distribution models considered in this work: SPSC, SPAC, and ASYM.

Illustrative V3​ and V4​ envelopes at electrode “Y,” simulated using the SPSC model, are shown in Figure 10C (top trace) alongside experimental data (bottom trace) for comparison. Simulations spanning the entire tracking dataset are plotted as solid lines, while validated frames (Figure 9C) are represented by circles. Simulated data were normalized to match the experimental amplitude to facilitate visual comparison.

The overall appearance of the simulated data qualitatively matches the experimental envelopes. Since the SPSC model is perfectly symmetric, it is incapable of reproducing the experimental asymmetry of amplitude excursions between V3​ and V4​ observed in the data (EXP envelopes in Figure 10C). However, the simulation effectively captures most envelope oscillations, as well as their relative amplitudes and polarity reversals throughout the experiment. Moreover, simulated envelopes derived from long sequential, non-validated tracking data exhibit oscillations (details a and b) that were already present in the experimental frames, albeit at a much smaller amplitude. This indicates that small body oscillations can be wrongly magnified by the tracking algorithm when the fish’s projection is captured against aquarium corners across multiple camera views. This may lead to the misidentification of skeletal segments.

Simulated data from all models were rescaled by constant factors to match the peak-to-peak amplitudes of the experimental envelopes’ oscillations at each electrode. This allowed for statistical quantitative analysis and comparison of results. It also enabled the inference of the source charge magnitudes required by each model to best reproduce the experimental envelopes (in place of the initial ±1 μC values). Table 1 shows the magnitude values of the source charges obtained by fitting the simulations to the experimental data. Q3​ represents the magnitude of the source charge for the V3​ wave component, while Q4​ corresponds to V4​. While the SPSC model is constrained by the condition Q3​ = Q4​ due to its inherent charge symmetry, Q3​ and Q4​ were independently fitted in the other models.

**Table 1:** Optimized source charge magnitudes (Q3​ and Q4​) obtained by fitting the simulated envelopes of each model to the experimental data.

Model	Q3 [pC]	Q4 [pC]
SPSC	75	75
SPAC	100	60
ASYM	233	222

Charges are in units of pico Coulombs.

**Figure 11:**
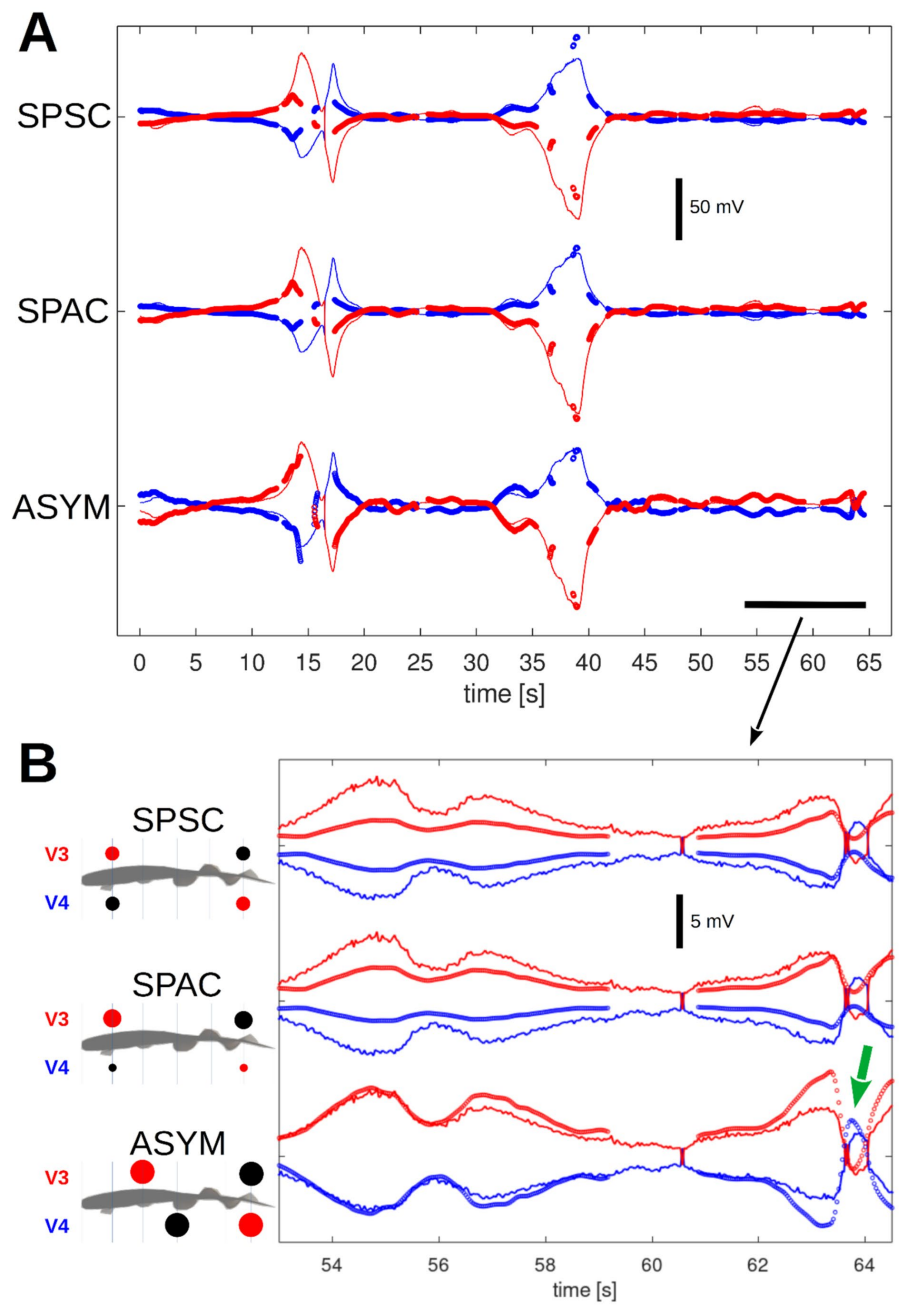
Envelope simulations at electrode X. Experimental data (thin solid lines) and simulation results (densely plotted small circles) are shown for V3 (red) and V4 (blue). Insets depict the fish with disks representing charge positions along the EO at V3 and V4, where disk color and disk size represent polarity (red: positive; black: negative) and relative charge magnitude (Table 1), respectively. **(A)** Full duration. **(B)** Magnified view of the region marked in **(A)**. The green arrow indicates envelope polarity reversals captured exclusively by the ASYM model, highlighting the role of source spatial asymmetry.

Q3 and Q4 correspond to peak charge accumulations at V3​ and V4, and are expected to exhibit comparable magnitudes. This occurs because they are driven by the same underlying electrophysiological mechanism of charge displacement along the EO. The SPSC model fails to reproduce the asymmetric behavior of the experimental V3​ and V4​ envelopes (Figure 11A). In the SPAC model, Q3​ and Q4​ are allowed to vary independently to better fit the data. Nevertheless, they ultimately diverge significantly (by approximately 40%) in an attempt to compensate for the experimental asymmetry through magnitude alone. In contrast, the ASYM model results in independently adjusted very similar values for Q3​ and Q4​ (ΔQ < 5%), as theoretically expected. This suggests that the experimental asymmetry is more accurately captured by accounting for the spatial asymmetry of the charge distribution rather than by varying the charge magnitudes between V3​ and V4​.

Figure 11 presents the simulation results (small circles; validated tracking data only) for the SPSC, SPAC, and ASYM models at electrode X, superimposed on the experimental envelopes (solid thin lines). As expected, Figure 11A shows that the SPSC model fails to accurately capture the asymmetry of the experimental data. This is evident near t = 38 s, where the blue simulation circles overshoot the experimental V4​ envelope, while the red circles undershoot V3​. Although the SPAC model shows improved performance at t = 38 s compared to SPSC, both models fail to fit the experimental envelopes around t = 15 s. Notably, the ASYM model exhibits a particularly poor fit at t ≈ 2 s. Visually, all models captured the polarity reversions occurring throughout the experiment in a similar fashion. Results obtained for all models and electrodes, in comparison with experimental data, are shown in Supplementary Figures S2–S4.

A zoomed view of the data around *t* ≈ 60 s is provided in Figure 11B to examine model behavior at low amplitudes. For *t* < 62 s, the ASYM model demonstrates significantly better performance than the other models; however, its accuracy deteriorates after t > 63 s. Nevertheless, in the vicinity of *t* = 64 s, a sequence of two polarity reversions occurs in the experimental envelopes (indicated by the green arrow), which is reproduced exclusively by the ASYM model. This serves as further evidence that spatial asymmetry in the source charge distribution plays a key role in shaping the envelopes’ dynamics.

### 3.4 Models’ pulse asymmetry

To assess the pulse asymmetry produced by the models and compare it with experimental results, simulations of the V3​ and V4​ components were conducted. A static, 16-cm-long virtual fish, comprising two aligned segments to match the length of the experimental specimen, was positioned at the geometric center of the simulation tank. Pairs of opposite electric charges (±Q3​ or ±Q4​) were placed along the skeleton according to each model, with magnitudes derived from fitting simulated envelopes to experimental data (Table 1). Subsequently, the electrical potential difference was calculated between virtual electrodes placed 1 cm from the head and tail, replicating the experimental head-to-tail pulse measurement. These simulation results are presented in Table 2.

**Table 2:** Summary of asymmetry evaluation and statistical results for the entire dataset.

Model	Asymmetry Δ%	Sum of squared errors (SSE) [V^2^]	Sum of absolute errors (SAE) [V]	Q-Q plot average model mismatch (x10^−3^)
IDEAL	–0.21	0	0	0
SPSC	0	0.3561	58.5	2.448
SPAC	–0.405	0.2606	53.1	2.179
ASYM	–0.193	0.2106	45.2	1.489

IDEAL represents the experimental data used as reference for comparison with the model’s results. Δ% is the asymmetry V3/V4. SSE and SAE represent the results of the time-based analysis, while quantile analysis results are expressed as the average model mismatch.

The pulse asymmetry in the SPSC model is zero, a direct consequence of the inherent spatial and temporal symmetry of its charge distribution. As discussed in the fitting analysis, the SPAC model attempts to account for experimental envelope asymmetry solely by varying the magnitudes of Q3​ and Q4​. However, because its source positions remain as symmetric as those in the SPSC model, this approach yields a pulse asymmetry of −40.5%, significantly diverging from experimental observations. In contrast, the ASYM model produces an asymmetry value of −19.3%—remarkably close to experimental results—while maintaining nearly identical magnitudes for Q3​ and Q4​.

### 3.5 Comparative analysis of the results

All statistical analyses are based on a representative dataset obtained from a single animal and should simply be considered a comparative fit analysis between models in our proof-of-concept study. The final simulated envelopes for each model after charge fitting for amplitude matching were quantitatively compared to the experimental envelopes. The comparison was done by calculating both the sum of squared errors (SSE) and the sum of absolute errors (SAE). A quantile-quantile analysis was also performed to compute the average model mismatch, defined as the area between the Q-Q plot curve and the reference line (Supplementary Figure S5). The results are presented in Table 2.

**Figure 12:**
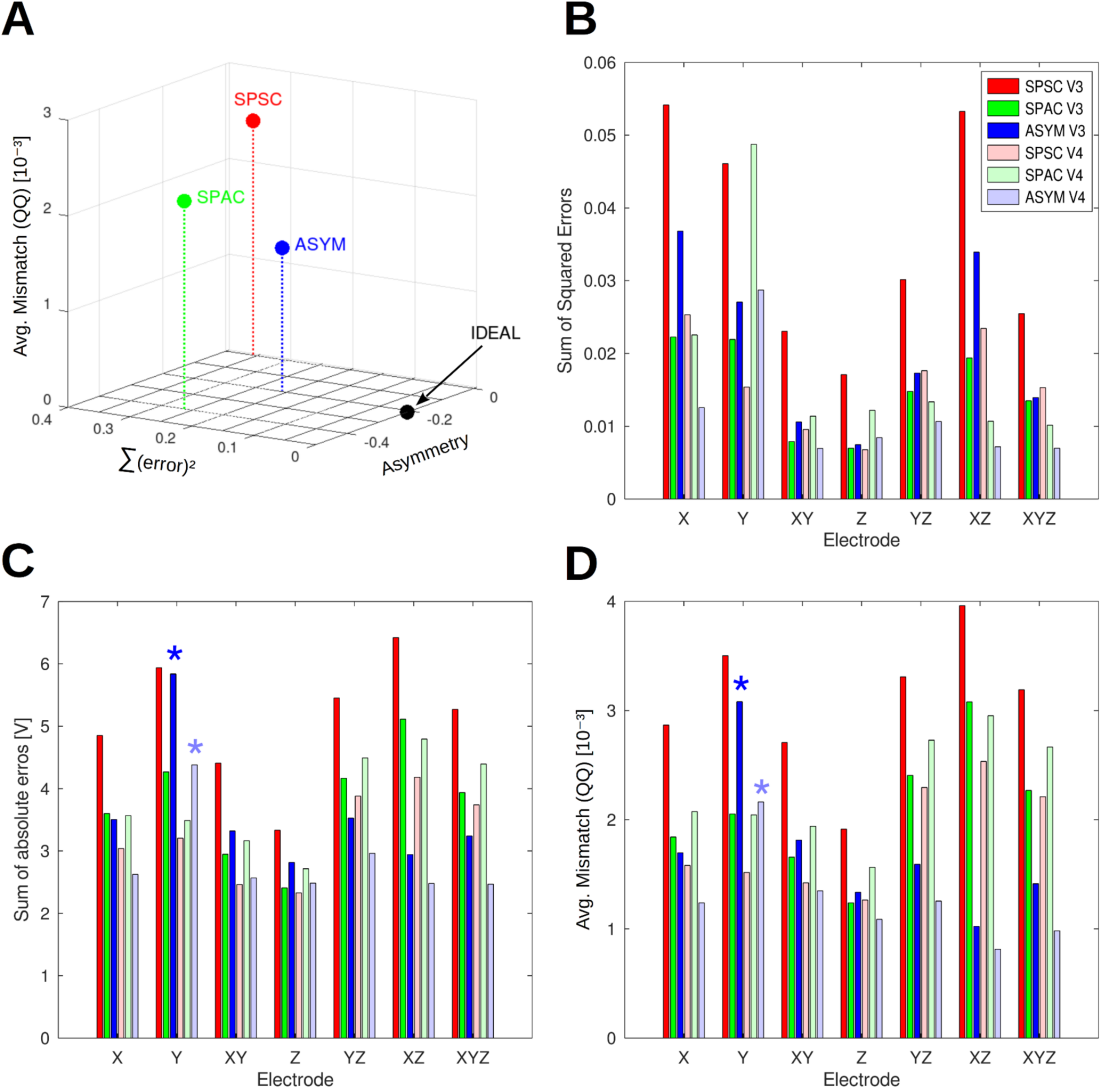
Summary and detailed assessment of models’ performance. SPSC, SPAC, and ASYM models are represented in red, green, and blue, respectively. **(A)** Head-to-tail asymmetry, total SSE, and aggregate Q-Q mismatch for all models compared against ideal results. **(B–D)** Detailed performance metrics categorized by electrode and envelope over all validated frames; the inset in **(B)** provides the color legend used across all panels. **(B)** Sum of squared errors (SSE). **(C)** Sum of absolute errors (SAE). **(D)** Average Q-Q plot model mismatch. While the ASYM model most closely approximates the ideal results, opportunities for further model refinement clearly remain. Blue bars marked with asterisks indicate poor ASYM results at electrode Y for both SAE **(C)** and average Q-Q mismatch metrics **(D)**.

Results accumulated over all valid frames in the dataset indicate that model performance improved as more realistic structural details were incorporated (from top to bottom of Table 2). The SPSC model yielded the poorest results, as it most closely approximates a classical dipole. Its only difference from the dipolar standard is the variable distance between charges, enabled by the rotation of skeleton segments at the mid-body joint. Permitting distinct charge magnitudes at V3​ and V4​ in the SPAC model led to better results. Finally, our comparative fit analysis showed that the ASYM model–that incorporates asymmetry in charge positions– exhibited the best performance. To illustrate these findings while simultaneously evaluating the reproduction of head-to-tail asymmetry, the results of each model were plotted against ideal values, as shown in Figure 12A. More detailed performance metrics are shown in Figures 12B–D, which display the SSE, SAE, and average Q-Q mismatch for each model, categorized by electrode and envelope.

The ASYM model results most closely approximate the ideal values (Figure 12A) in our representative dataset. Because SSE is highly sensitive to outliers, the detailed results in Figure 12B highlight the poor performance of the SPSC model, which stems primarily from localized overshoots and undershoots of the envelopes at high amplitudes—particularly when the fish approaches the “REF” electrode [see Q-Q plots (Supplementary Figure S5) and simulated envelopes (Supplementary Figures S2–S4)]. In contrast, because SAE is more robust to outliers, the poor results obtained for the ASYM model at electrode Y (Figure 12C, blue asterisks) indicate a more consistent misalignment with the experimental envelopes; this is further confirmed by the Q-Q average model mismatch metrics (Figure 12D).

The dependence of pulse-effect spatial distribution on the chosen charge-distribution model reveals the inherent limitations of using a single pair of electrodes as an “artificial fish.” These are intended to electrically interact with freely moving animals (Forlim and Pinto, 2014; Waddell and Caputi, 2021). Our results suggest that employing two separately driven pairs of wires (based on the ASYM model) produces quantitatively more realistic spatial effects than models utilizing a single pair, such as those based on the SPSC or SPAC configurations. This improved spatial accuracy might prevent the live fish from miscalculating the distance or orientation of the artificial agent, thereby facilitating more naturalistic social interactions.

### 3.6 From envelope mismatch analysis to charge-distribution refinement

**Figure 13:**
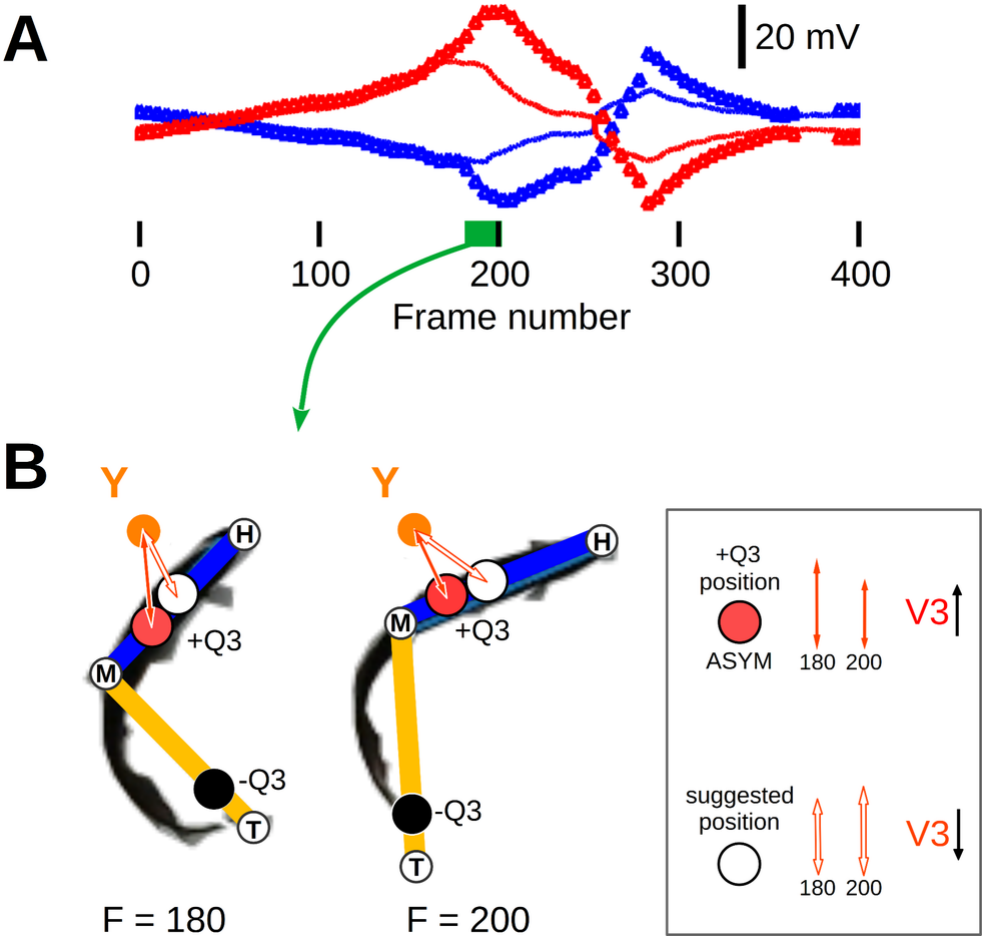
Detailed analysis of envelope misalignment and tracking images. **(A)** Frame sequence of V3 (red) and V4 (blue) experimental envelopes (thin continuous lines) compared to ASYM model simulations (dots) for electrode “Y” (zoomed from Supplementary Figure S4). Previously well-aligned simulated and experimental envelopes diverge after frame 180 (green horizontal bar), highlighting a localized breakdown in model accuracy. **(B)** Raw fish images and electrode “Y” position extracted from Video S1, frames 180 and 200 (Camera 3, top view); the “REF” electrode (not shown) is located at a large vertical distance. Skeleton segments (blue and orange) are superimposed on the images to indicate head (H), mid-body (M), and tail (T) points identified by the tracking algorithm. The position of the +Q3 charge in the model is represented by a red circle at V3 in both frames, while the −Q3 charge is represented by a black circle. The white circle represents a hypothetical correction to the charge position. Red solid arrows indicate the distance from electrode “Y” to the current charge position, whereas red arrows (white fill) indicate the distance to the corrected position. Inset: Arrows from both frames are rotated and pairwise aligned, maintaining their relative lengths. The inset demonstrates that while the distance from the electrode to the current charge position decreases from frame 180 to 200—leading to an erroneous increase in V3 amplitude—the distance to the corrected position increases, thereby decreasing the V3 envelope and matching the experimental dynamics.

To further investigate the anomalies in the ASYM model at electrode “Y” revealed at the statistical analysis, we examined the full time-series plots of the simulated and experimental V3​ and V4​ envelopes (Supplementary Figure S4). This allowed us to identify the specific temporal regions, framewise, where the simulated envelopes deviated most significantly from the experimental data (Figure 13A). Near frame 200 (*t* ≈ 6.7 s, indicated by a green horizontal bar), the experimental V3​ (red) and V4​ (blue) envelopes (thin continuous lines) are observed to decrease in amplitude across successive frames. Conversely, the ASYM simulated envelopes (dots) diverge, exhibiting the opposite trend by showing a progressive increase in amplitude over the same interval.

Video inspection revealed that during this stage of the experiment, the fish restricted its movement primarily to a nearly horizontal plane. Consequently, images from Camera 3 (top view) alone were sufficient to illustrate the body configuration. Under these conditions, the projected distance between the fish and electrode “Y” in this perspective corresponds directly to the true physical distance, enabling an accurate assessment of the geometric mismatch. This specific interval corresponds to the onset of the 3D trajectory previously plotted in Figure 8 (see Supplementary Video S1 starting at 6 s). Figure 13B shows the fish images and electrode Y positions extracted from frames 180 and 200 (around *t* = 6.3 s), with superimposed skeleton segments and the charge distribution (red circle = positive charge; black circle = negative charge) for V3​ according to the ASYM model. The “REF” electrode (not shown) is located at a large vertical distance. Since the envelopes are simulated by applying Coulomb’s law, the primary contribution to V3​ is determined by the inverse of the distance between the positive charge and electrode “Y” (Figure 13B —solid red arrow).

In the ASYM model, the positive charge for V3​ was initially positioned at one-third of the head-to-mid-body segment, measured from the midpoint. Consequently, as the fish moved, the distance between this charge and electrode ‘Y’ decreased between frames 180 and 200. This caused the simulation to predict an erroneous increase in V3​ amplitude. However, as illustrated by the white circles in Figure 13B and its inset, shifting the positive charge to a more rostral position would have increased its distance to electrode ‘Y’ over that same interval.

Furthermore, the overly caudal placement of the positive charge likely artificially increased its distance from the electrode at other points. This resulted in a fitted magnitude for Q3​ that was larger than physiologically necessary. Implementing this geometric correction will likely yield smaller, more realistic magnitudes for both Q3​ and Q4​ while maintaining the same signal fidelity.

Moreover, this finding also reveals how spatial errors can translate into temporal discrepancies. The suggested rostral correction of the positive charge position would also account for the reduction or elimination of the polarity reversion delay observed between the experimental and simulated envelopes near frame ≈ 260. As the fish continues its turn, it reaches an orientation where the positive and negative charges are equidistant from electrode Y, resulting in a zero-amplitude (null) envelope. Because the positive charge in the ASYM model is positioned more caudally than in the actual fish, the model requires a greater angular rotation to reach this equidistant state. This geometric discrepancy generates the temporal delay observed in the simulated reversion relative to the experimental ground truth.

This refinement was not explored in further detail. But, we estimate the precision in correcting the charge positions to be on the order of one-tenth of the segment length (i.e., ~5 mm). The primary challenge lies in the degree to which each body section approximates a straight segment. This geometrical fitting constraint is particularly evident in the caudal region, given its inherent flexibility and intense electrical activity. Moving forward, we intend to replace these linear segments with circular arcs of constant arc length and variable curvature to better replicate the fish’s pose at each frame.

While we have presented a simple illustrative case here, the same sequence of diagnostic procedures can be systematically applied to refine the negative charge position for V3​, as well as all charge coordinates for V4​. Although the utility of these methods for fine-tuning models was a significant outgrowth of the project’s execution—demonstrating broad applicability and internal consistency—a full exploration of model development and refinement remains outside the scope of this work.

## 4 Conclusion

Long, continuous, simultaneous video and electrophysiological recordings are essential tools in neuroethological studies to relate neural dynamics to associated behavior (Datta et al., 2019). However, their implementation is difficult and in most cases involves some degree of invasiveness. For this reason, in many studies the association is performed separately, using video tracking on the one hand and electrophysiological recordings during fictive behavior on the other (e.g., Levi et al., 2004). Only in specific animal species, and under specific invasive protocols, are simultaneous video and electrophysiological recordings performed. Even under these conditions they are performed with limited capabilities due to synchronization challenges and constraints from the difference in video-tracking and electrophysiological temporal resolution (Etholm et al., 2010; Sattler and Wehr, 2021; Liu and Gluckman, 2026).

Weakly electric fish are animal models where noninvasive neuroethological studies can be implemented. In these animals, dual video and electrophysiological recordings provide the means to associate electrical and motor activity across various behavioral contexts (e.g., communication, electrolocation, spatial orientation, feeding, and social interactions). All behavioral contexts mentioned require highly precise spatio-temporal integration. Such dual recordings can enhance our understanding of neural encoding. By identifying specific signatures and disentangling communication from electrolocation signals, they can provide direct associations between electrical activity and behavior. Since multiple natural behavioral contexts occur exclusively at night or in turbid waters, the ability to track the position of animals in the absence of light provides novel opportunities for neuroethological studies. The proposed methodology represents a critical first step toward gathering both signal and positional data without the necessity of continuous video recording.

We presented an open-source solution that should be relatively easy to implement in laboratories working with electric fish. It is based on a combination of integrated techniques: multielectrode array recordings with simultaneous multi-camera video, deep learning-based tracking, and 3D tracking-based simulations of electric potentials. In this proof-of-concept study the framework was applied to correlate the position and pose of an electric fish with the pulses detected in an array of electrodes surrounding the freely moving animal. Each component of the method was described in detail and validated using representative case-study data.

Despite the use of off-the-shelf USB webcams, this approach provides a high temporal resolution often absent in other neuroethological techniques. Furthermore, its fast performance enables triggering closed-loop stimulation, as an alternative/complement to video-triggered stimulation in electric fish (Chamorro et al., 2012).

Applying nonlinear amplifiers allowed for the detection of all pulses regardless of the fish’s position and enabled the restoration of the original signal at each electrode. No signal saturation was observed across the entire dataset, with recorded values remaining strictly between −9 V and +9 V (within the ±10 V limits). The system exhibited a total noise of approximately 1 mV in the reconstructed signals, reflecting primarily the electromagnetic pick-up and electronic noise accumulated along the entire signal chain. Pulse amplitudes consistently reached tens of millivolts in at least one electrode. This ensured a high signal-to-noise ratio for all recorded data, likely due to the use of unity-gain buffers (active electrodes). Total noise could be reduced by using larger carbon electrodes instead of stainless steel thin wires. The latter have higher impedance and are less chemically inert. However, they were chosen for their practicality in this work. They could be easily inserted at any point through the silicone sealant between the glass walls. This facilitates the conversion of virtually any common glass aquarium into an experimental chamber. Nevertheless, an improved apparatus utilizing carbon electrodes as sensors is under development.

The recovery of the original signals permitted the construction of pulse envelopes based on the actual amplitudes of wave components V3​ and V4​ of the EOD across the array. EOD pulses contain fast dynamic components (on the kHz scale), whereas the envelopes exhibit much slower, motion-modulated dynamics that align with the temporal resolution of the video cameras (30 FPS). Furthermore, the average fish pulse rate typically exceeds 30 pulses per second, ensuring an adequate density of envelope data paired with the tracking.

Manually comparing the experimental envelope data with the fish position in representative situations revealed a strong agreement between the sequence of positions and poses of the fish relative to an electrode and the dynamic electrical signals recorded at that location.

Quantitative analysis of the deep learning-based video tracking procedures demonstrated a significant overall precision of 0.8 mm per coordinate. It can be further improved in the future through the addition of more cameras to the setup. Manual validation confirmed that key skeleton points were correctly localized in 81% of frames across all camera views. Nevertheless, a redesigned experimental tank and an improved array of electrodes are currently under development to mitigate visual artifacts and further enhance tracking and electrical signal accuracy.

Tracking-based envelopes were simulated using three simplified models of charge distribution along the EO. In these models, a pair of opposite charges was chosen to represent the positions of two centers of charge within the EO. Thus, simplifying the real continuous charge distribution at the peak of each wave component, V3​ and V4​. These models represented distinct physical perspectives: the SPSC, closely analogous to an electric oscillating dipole; the ASYM, which follows a more anatomically inspired framework; and the SPAC, which serves as an intermediary approach.

Simulations of V3​ and V4​ envelopes were carried out using Coulomb’s Law applied to the two source charges and their 52 image charges to account for tank interface effects up to the third order. Quantitative comparisons demonstrated an increasingly better fit across the models, with ASYM providing the highest accuracy in this representative case-study dataset. These results may also be relevant for bio-robotic interaction. In particular, they suggest that a simple dipole electrode pair may not reproduce the spatial signal structure of a real fish well enough, whereas a four-wire design may provide a more realistic alternative.

The similar fitted values of Q3 and Q4 in the ASYM model suggest that a common underlying mechanism may shape charge displacement in both wave components. Such consistency remains observable despite the simplification of the EOD’s complex space–time dynamics. First, by replacing continuous temporal evolution with instantaneous states at the peak of the V3 and V4 components, and subsequently, by localizing the distributed charges into two discrete points while maintaining the overall electrical neutrality of the EO.

Evaluating the different charge distributions served to validate the system’s robustness and accuracy. Furthermore, the capacity to iteratively refine these EO models emerged as another methodological contribution of the project. Together, these results support the reported methods for obtaining integrated spatial and electrical data.

Looking ahead, the system could be adapted into a platform for studying the internal structure of the EO. Expanding the number of simulated source charges and iteratively refining their spatial distribution would offer a novel, non-invasive window into the bioelectrical complexities of the electric organ.

In the future, several aspects of our work can be generalized to some other pulse- or wave-type electric fish, especially if they have EOs that extend along almost the entire length of the body, such as in *Gymnotus carapo*. However, its applicability will likely be limited in cases where the EO is more localized, such as in *Gnathonemus petersii.* The methodology should also be scalable to multiple animals, provided their electrical and motor behaviors can be recorded simultaneously during natural exploratory or complex social interactions.

Moreover, obtaining these integrated data sets is a first step toward training precise, video-free 3D tracking machine learning methods. Such methods represent a promising route for analyzing fish kinematics and naturalistic conspecific interactions in undisturbed, dark, or turbid environments.

## Data Availability

The original contributions presented in the study are included in the article/Supplementary material, further inquiries can be directed to the corresponding author/s.

## Footnotes

1
www.sfn.org

2
https://octave.org
