# Supplementary material for: Integrating deep learning 3D tracking and biophysical EOD modeling for precise, noninvasive computational neuroethology in freely swimming weakly electric fish
Source: Front Comput Neurosci. 2026 Jun 24;20:1810975. doi: 10.3389/fncom.2026.1810975 (PMC13341423; doi:10.3389/fncom.2026.1810975)
Supplement: Supplementary file 1 [file Data_Sheet_1.docx]

**Supplementary Material**

**Video_S1.mp4**


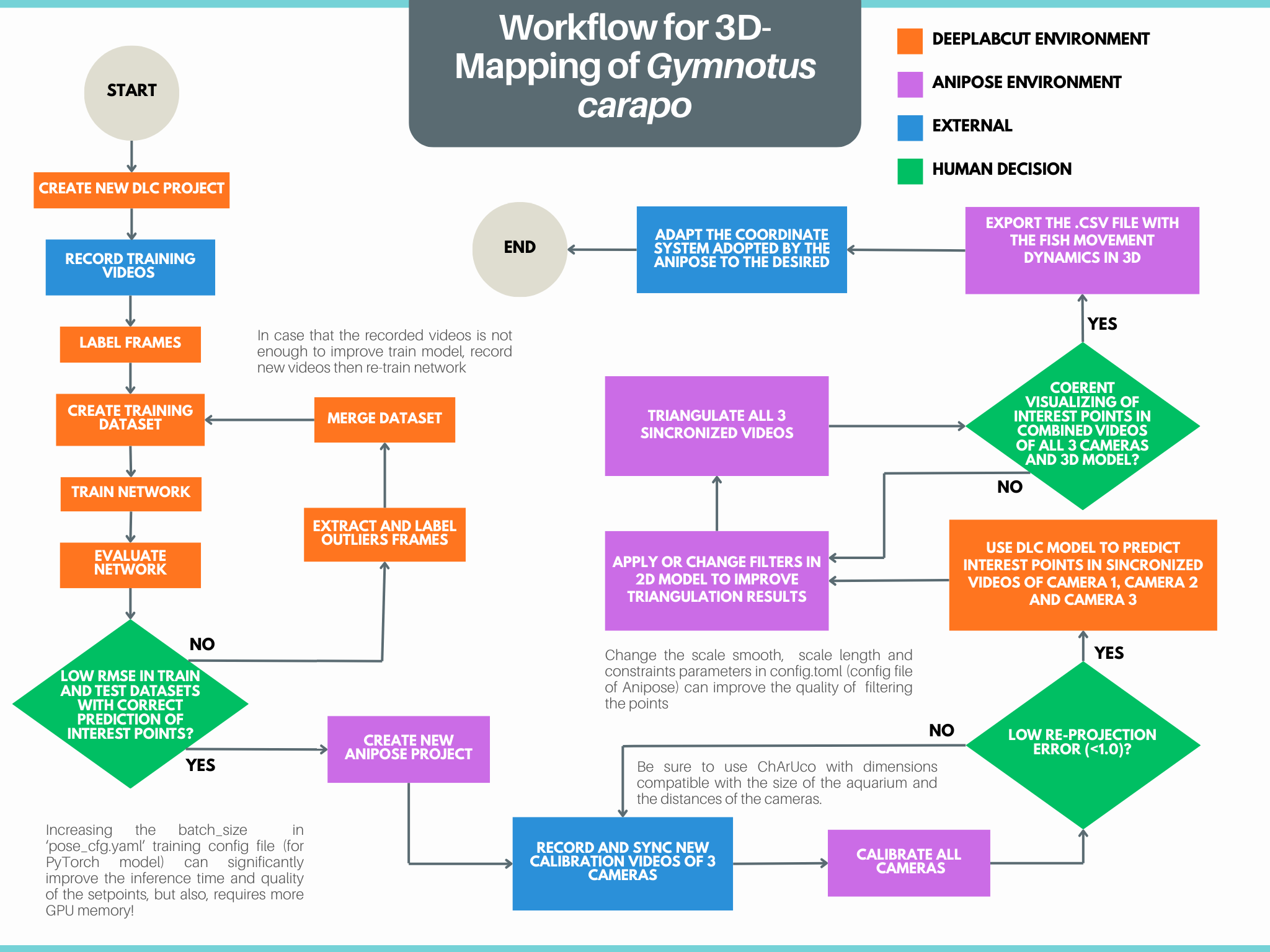


**Figure S1 - Workflow for the 3D tracking of *Gymnotus carapo*.** The process began with fine-tuning a neural network into DeepLabCut (DLC) to identify the fish's head, mid-body, and tail using 2D manually labeled frames from multiple animals and aquariums. Subsequently, after reducing the RMSE in both the train and test datasets being careful to avoid overfitting, an Anipose project was initialized, and the three cameras were calibrated using a ChArUco board submerged in the tank, obtaining the calibration matrix with the intrinsical and extrinsical parameters for the experimental setup of cameras. The fine-tuned DLC model extracted anatomical points from the synchronized camera views, which were then filtered and triangulated by Anipose. Finally, a change-of-basis matrix was applied to the trajectories to transform the native software coordinates (based on the center of Camera 1 sensor) into the experimental tank metric coordinate system (X,Y,Z).


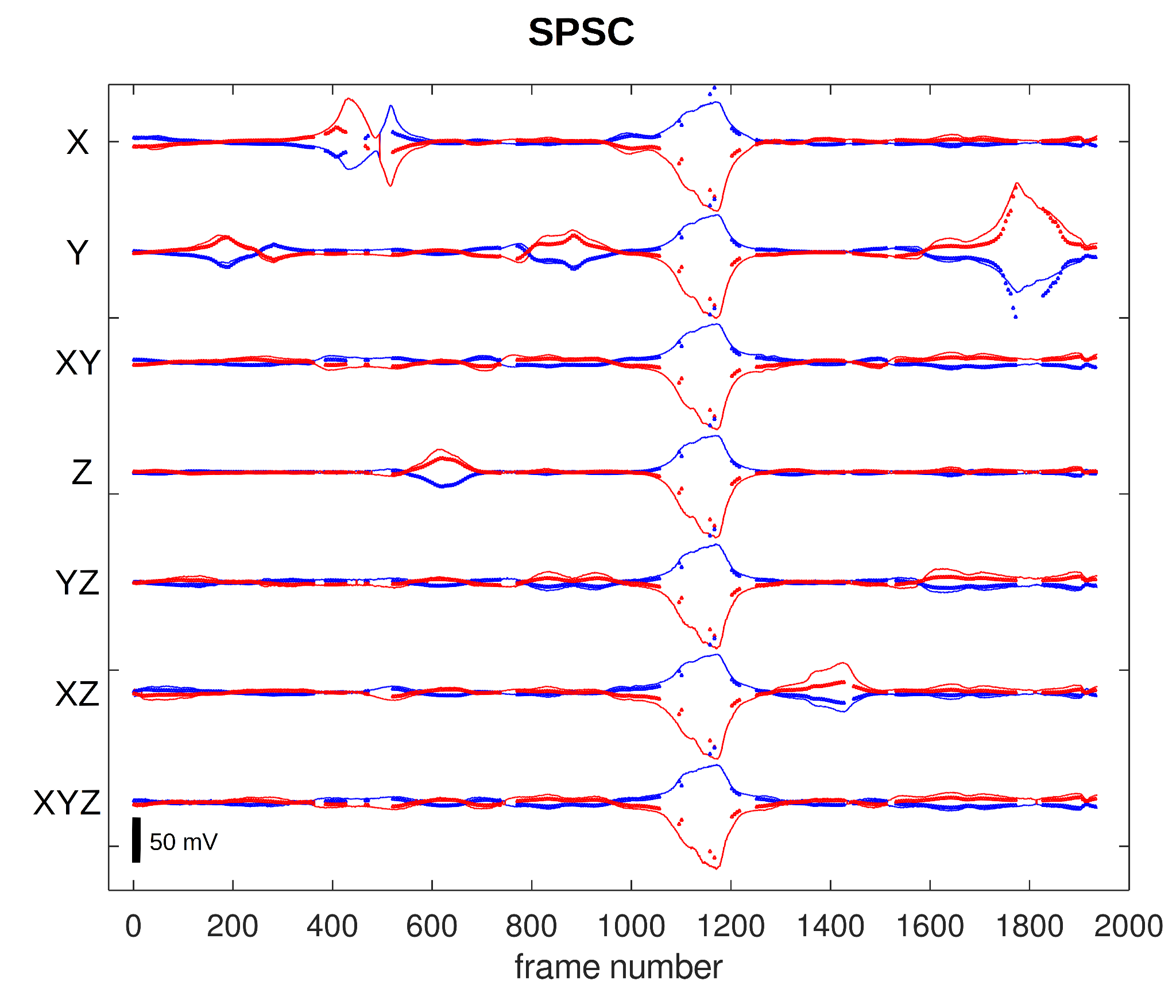


**Figure S2 - Envelope simulations for the SPSC model.** Simulation results (densely plotted small circles) for V3​ (red) and V4​ (blue) are superimposed on experimental data (thin solid lines).


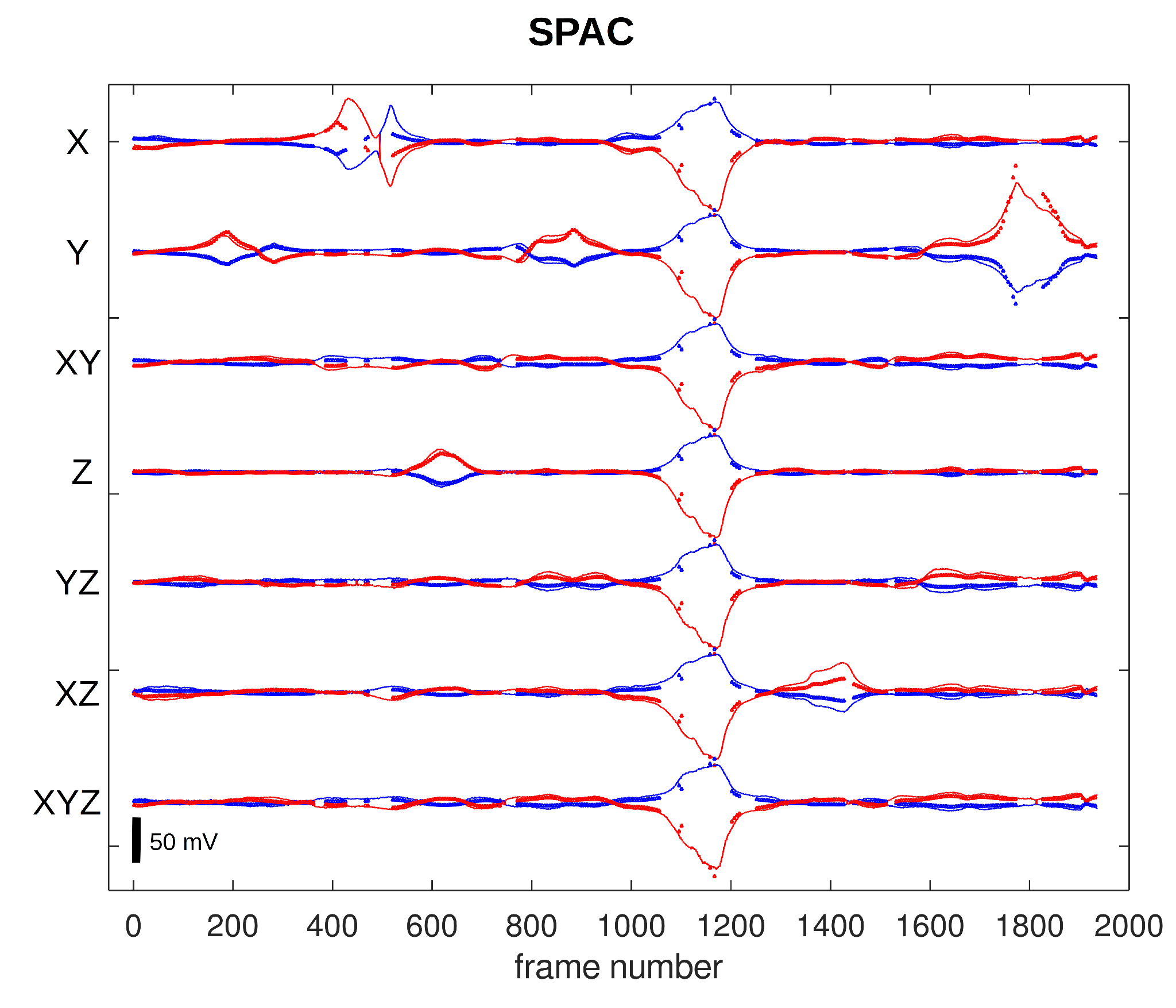


**Figure S3 - Envelope simulations for the SPAC model.** Simulation results (densely plotted small circles) for V3​ (red) and V4​ (blue) are superimposed on experimental data (thin solid lines).


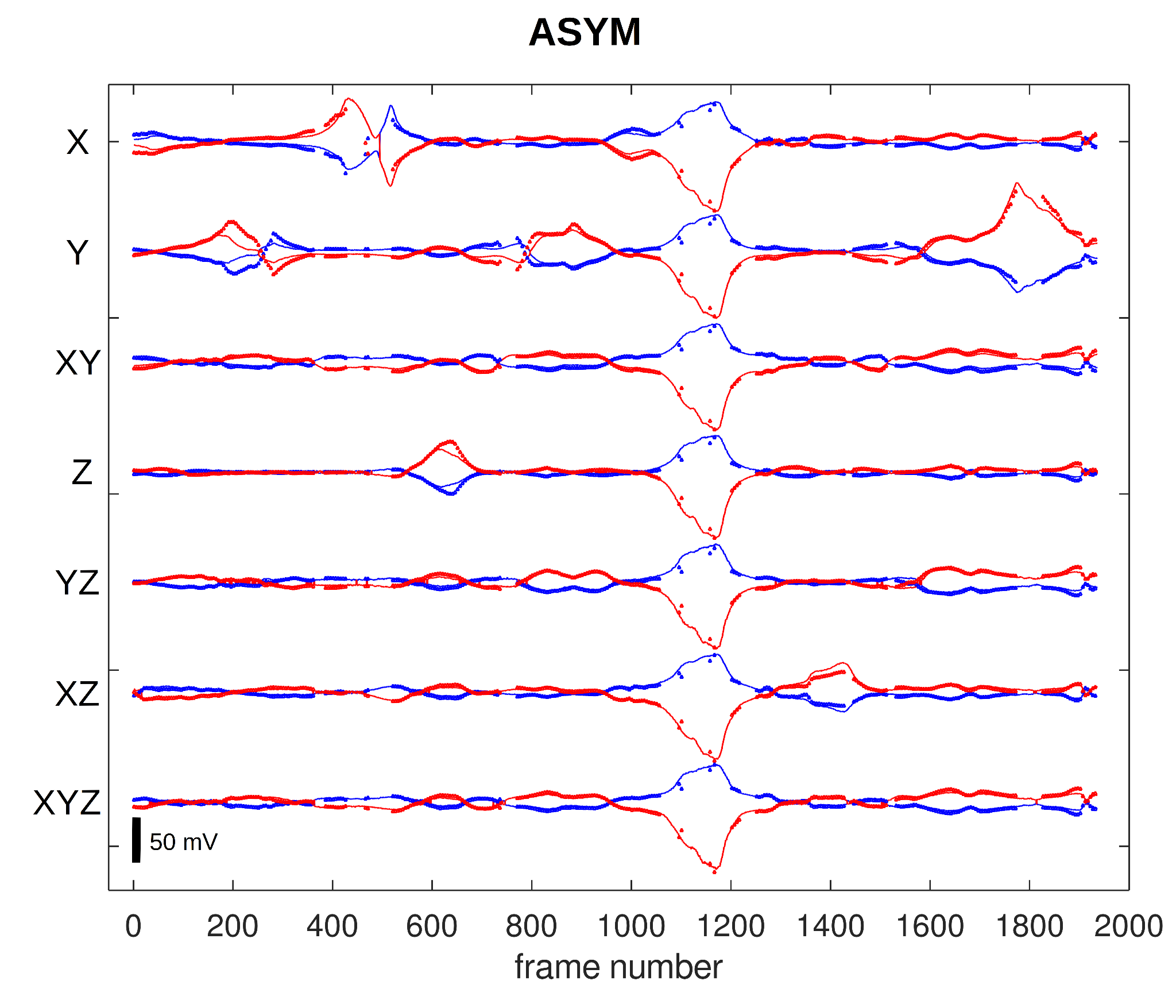


**Figure S4 - Envelope simulations for the ASYM model.** Simulation results (densely plotted small circles) for V3​ (red) and V4​ (blue) are superimposed on experimental data (thin solid lines).

**
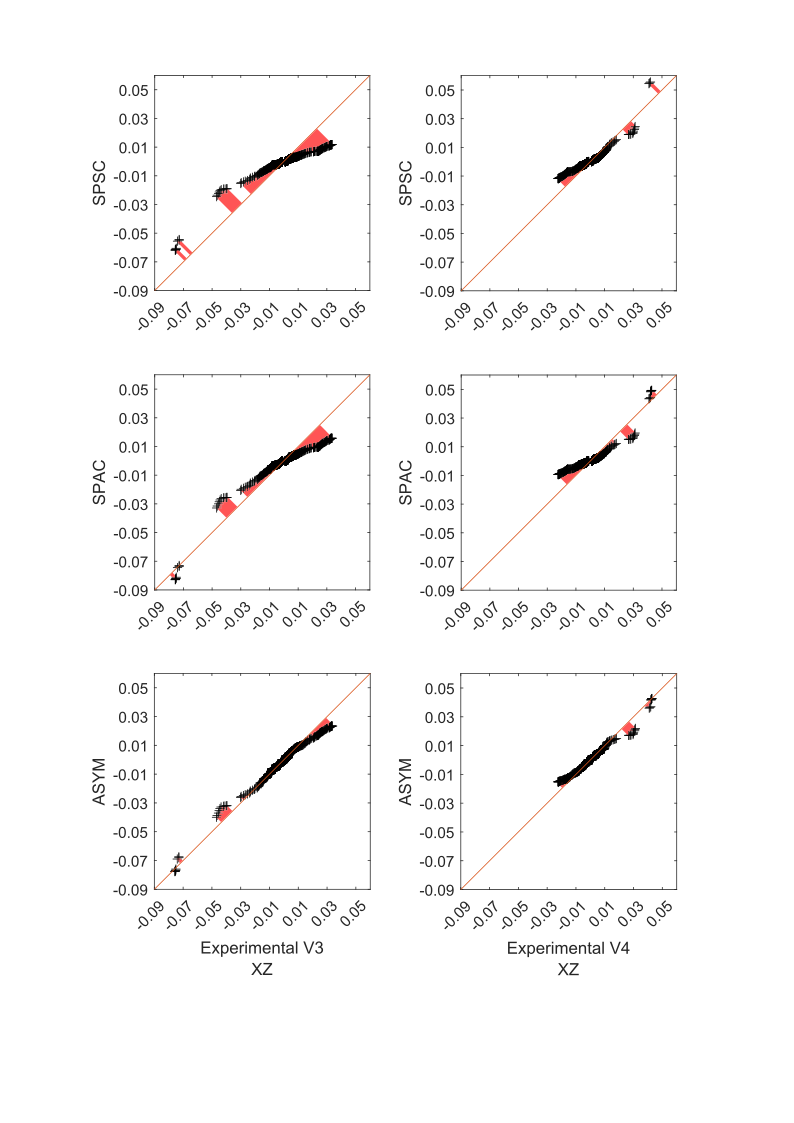
**

**Figure S5 - Quantile-quantile (Q-Q) plots for electrode XZ across all models.** The distributions of the modelled and measured amplitude of the electric signal are sorted and plotted against each other. The red line represents the identity line (y = x, slope = 1); points lying exactly on this reference line indicate that the modeled and measured amplitudes are identical. To quantify model mismatch, we calculated the average area between the reference line and the data points (highlighted in red). Corresponding numerical results are provided in Table 2 and Fig.12D in the main text.
